# Unveiling nanoscale optical signatures of cytokine-induced β-cell dysfunction

**DOI:** 10.1038/s41598-023-40272-9

**Published:** 2023-08-16

**Authors:** Licia Anna Pugliese, Valentina De Lorenzi, Mario Bernardi, Samuele Ghignoli, Marta Tesi, Piero Marchetti, Luca Pesce, Francesco Cardarelli

**Affiliations:** 1 NEST Laboratory - Scuola Normale Superiore, Piazza San Silvestro 12, Pisa, Italy; 2 Department of Clinical and Experimental Medicine, Islet Cell Laboratory, University of Pisa, Pisa, Italy

**Keywords:** Biophysics, Nanoscale biophysics, Cellular imaging, Diabetes

## Abstract

Pro-inflammatory cytokines contribute to β-cell failure in both Type-1 and Type-2 Diabetes. Data collected so far allowed to dissect the genomic, transcriptomic, proteomic and biochemical landscape underlying cytokine-induced β-cell progression through dysfunction. Yet, no report thus far complemented such molecular information with the direct optical nanoscopy of the β-cell subcellular environment. Here we tackle this issue in Insulinoma 1E (INS-1E) β-cells by label-free fluorescence lifetime imaging microscopy (FLIM) and fluorescence-based super resolution imaging by expansion microscopy (ExM). It is found that 24-h exposure to IL-1β and IFN-γ is associated with a neat modification of the FLIM signature of cell autofluorescence due to the increase of either enzyme-bound NAD(P)H molecules and of oxidized lipid species. At the same time, ExM-based direct imaging unveils neat alteration of mitochondrial morphology (i.e. ~ 80% increase of mitochondrial circularity), marked degranulation (i.e. ~ 40% loss of insulin granules, with mis-localization of the surviving pool), appearance of F-actin-positive membrane blebs and an hitherto unknown extensive fragmentation of the microtubules network (e.g. ~ 37% reduction in the number of branches). Reported observations provide an optical-microscopy framework to interpret the amount of molecular information collected so far on β-cell dysfunction and pave the way to future *ex-vivo* and *in-vivo* investigations.

## Introduction

Pro-inflammatory cytokines (e.g., IL-1β, TNF-α, and IFN-γ) are well-characterized effectors of β-cell failure in both Type-1 and Type-2 Diabetes^1,2^. In the last decades of research, thanks to a combination of standard genomic, transcriptomic, proteomic, and biochemical approaches, the alteration of the molecular landscape induced by cytokines was quantitatively defined in β cells from human islets, mouse islets, and clonal lines^1–6^ From collected data, a few hallmarks of β-cell molecular response to cytokines emerge. First, by the analysis of proinsulin ‘interactome’, it was defined that even short-term exposure to pro-inflammatory cytokines reshapes proinsulin interactions with critical regulators of the secretory pathway in β-cells, leading to aberrant glucose-independent hormone secretion^7–10^. Worthy of note, at the same time, ELISA-based secretion assays demonstrated that cytokines impair glucose-stimulated insulin secretion (GSIS) thus pushing β cells towards a metabolic status of reduced responsiveness^5,11^. Second, compelling evidences demonstrated that increased intracellular oxidative stress follows β-cell exposure to pro-inflammatory cytokines^1,2^. In particular, recent findings suggest a role for nicotinamide adenine dinucleotide phosphate (NADPH) oxidases (Noxs) and inducible nitric oxide synthase [iNOS] in the generation and propagation of reactive molecules and metabolites leading to β-cell dysfunction^12^. Regard to this latter, while the involvement of the Endoplasmic Reticulum (ER) as central hub in integrating death/stress-inducing signals appears clear^13–16^, accumulating evidences suggest that mitochondria are additional key players in the process^4,17–21^. Indeed, Barbu and co-workers showed that cytokine-induced β-cell failure is preceded by disruption of mitochondrial membrane potential in rat RINm5F cells^22^; similarly, Grunnet and co-workers found that cytokines are able to induce mitochondrial stress and cytochrome-c release, with subsequent activation of caspase-9 and -3 and induction of DNA fragmentation in human and rat islets and in INS-1E cells^2^. Notwithstanding the efforts devoted to define the molecular landscape of β-cell response to cytokines, however, no report thus far complemented the acquired molecular knowledge with the direct observation, via high-resolution imaging, of cytokine-exposed β-cells. In fact, Transmission Electron Microscopy (TEM) was used to build a large-scale imaging database using tissues from both healthy and diabetic donors^23^, but with inherently poor molecular specificity. On the other hand, the exploitation of optical microscopy, which potentially holds high molecular specificity and high spatial resolution^24^, is still in its infancy in the study of β-cells and it has been used so far primarily to investigate ISG trafficking and β-cell response to glucose stimulation^25–27^ (for a detailed review of optical-microscopy-based investigations on β-cells, see Ref.^28^). Here we exploit a combination of state-of-the-art high-resolution optical-microscopy tools to directly observe the intracellular landscape of INS-1E β-cells exposed to IL-1β and IFN-γ pro-inflammatory cytokines for 24 h and recognize modifications of selected measurable parameters. On one hand, label-free fluorescence lifetime imaging microscopy (FLIM) of β-cell intrinsic signals was used to quantitatively probe either the balance between free and enzyme-bound NAD(P)H molecules and the intracellular accumulation of oxidized lipid species. On the other hand, fluorescence-based expansion microscopy (ExM)^29,30^ was used to achieve the spatial resolution (~ 50 nm in this study) needed to analyze the fine structural properties of the key subcellular targets, namely: mitochondria, insulin granules, actin filaments, and microtubules. It was found that 24-h exposure to IL-1β and IFN-γ is associated with a neat modification of the FLIM signature of cell auto-fluorescence due to the increase of either enzyme-bound NAD(P)H molecules and of oxidized lipid species. At the same time, ExM-based direct imaging unveiled that cytokines induce neat alteration of mitochondrial morphology (i.e. ~ 80% increase of mitochondrial circularity), marked degranulation (i.e. ~ 40% loss of insulin granules, with mis-localization of the surviving pool), appearance of F-actin-positive membrane blebs and an hitherto unknown extensive fragmentation of the microtubules network (e.g. ~ 37% reduction in the number of branches). Reported observations provide an optical-microscopy-based framework to interpret the amount of molecular information collected so far on β-cell dysfunction and pave the way for future *ex-vivo* and *in-vivo* investigations aimed at evaluating quantitatively the contribution of cytokine-induced β-cell alterations in Diabetes and their possible modification in response to drug treatments.

## Results

### Overall experimental workflow

**Figure 1:**
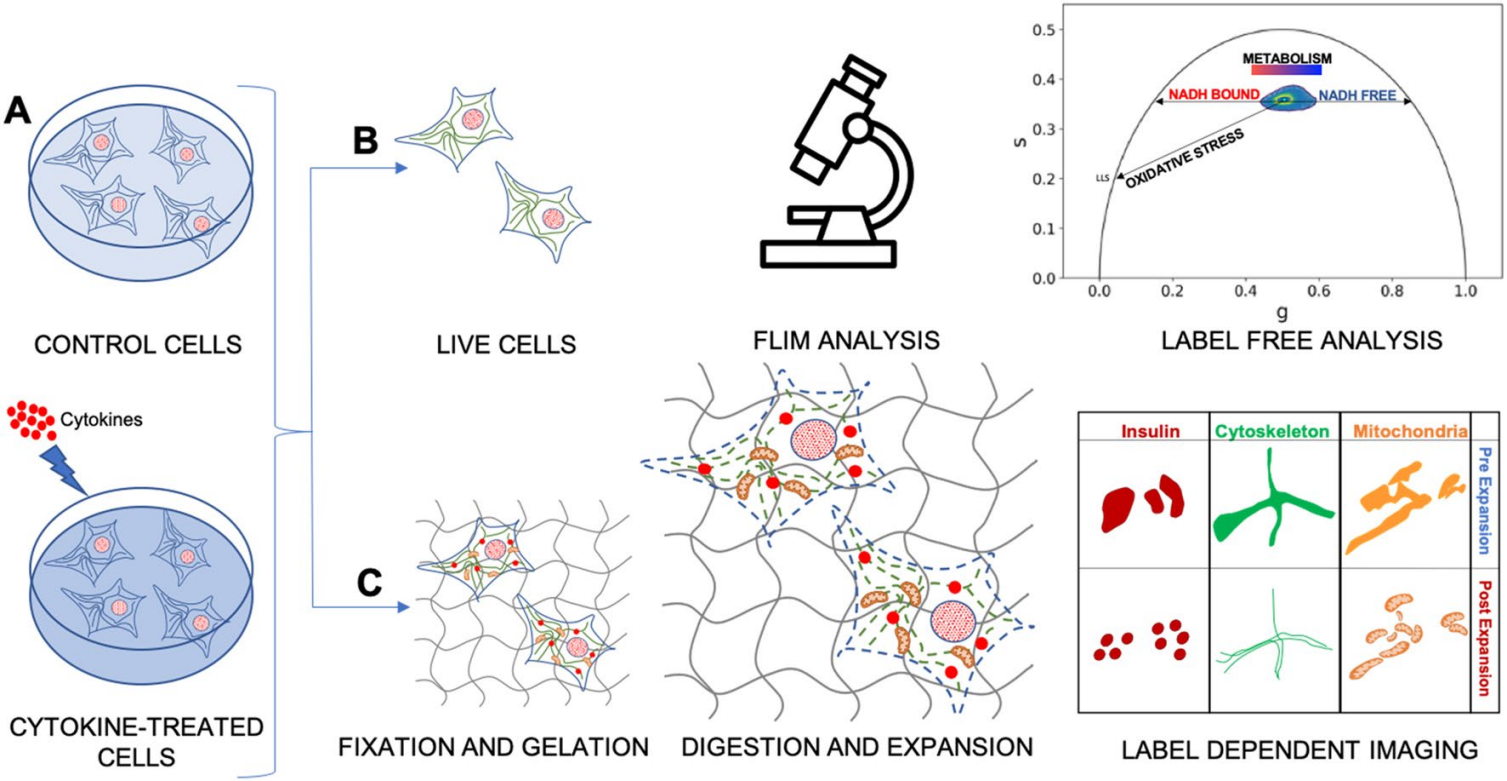
Schematic representation of the general workflow of our experiments. (**A**) INS-1E were plated and then incubated for 24 h in fresh complete medium or supplemented with cytokines. (**B**) A label-free analysis (FLIM) was performed on NAD(P)H signal to assess changes in cellular metabolism after cytokine treatment. The result is a phasor plot in which the cellular metabolism oscillate between the signal of NADH free, NADH bound and long lifetime species (LLS) exploiting their different lifetime. (**C**) Then, the specimens were chemically fixed, stained with fluorescence probes and antibodies, functionalized with AcX, gelled, and finally expanded. Cytokine-treated and control samples were stained for three subcellular structures (mitochondria, ISGs, and cytoskeletal) using ExM.

Figure 1 summarizes the general experimental workflow used in this study. Insulinoma 1E (INS-1E) cells share many characteristics with primary β-cells (e.g. glucose-sensing ability) and are therefore widely used as a β-cell model^31^ (Fig. 1A). On one hand, we exploited fluorescence lifetime imaging microscopy (FLIM) on live cells to assess the functional response to cytokines (Fig. 1B): indeed, FLIM performed on intrinsic cell autofluorescence (excited at 740 nm and collected in the 420–460-nm range) can be used to interrogate both cell metabolic response, in terms of the ratio between the free and protein-bound forms of NAD(P)H molecules^32–35^, and the possible emergence of ROS-dependent oxidative stress, in terms of lipid species with long characteristic lifetimes^36^. Both readouts were analyzed by means of the well-established, fit-free, and graphical phasor-based approach to FLIM^37^ (Fig. 1B, right panel). On the other hand, we used super-resolution fluorescence-based microscopy, in the form of Expansion Microscopy (ExM), to analyze the structural properties of selected subcellular targets in response to cytokines, namely: insulin granules, cytoskeleton (both microtubules and actin filaments), and mitochondria (Fig. 1C). To this end, cells were first chemically fixed using paraformaldehyde, glutaraldehyde, or a combination of both, to crosslink neighboring proteins^38^; next, cells were stained using conventional antibodies functionalized with fluorescent probes, optimizing the labeling protocol for each biological target to be investigated (see Methods for more details). According to ExM standard procedure, Acryloyl-X SE (AcX) molecular handle^39^ was covalently attached to virtually any of the primary amine groups of labels and biomolecules in the sample, enabling them to be anchored to the hydrogel. At this point, the specimen was soaked in a solution consisting of acrylamide, bisacrylamide, and sodium acrylate to generate a densely cross-linked gel throughout the specimen. Such a hybrid sample was finally placed in a digestion buffer (to split up the protein content), and expanded in distilled water by exploiting the highly charged nature of the polyelectrolyte backbone^30^. The final expansion factor (EF) was determined using cell size, and then confirmed by two independent markers, i.e. Phalloidin and TOM20, which label actin filaments and mitochondrial membranes respectively, and was found to be of 4.7 ± 0.4 folds (Fig. S1), a value that corresponds in our system to a new nominal resolution for imaging of 50 ± 8 nm (Fig. S2). In addition, the pre- and post-expansion confocal images of the same stained cells were compared, showing low distortion and a high structural similarity index (calculated as reported in Refs.^40,41^) for all the stained structures (total SSIM index = 0.73 ± 0.05) (Fig. S3).

### INS-1E cells exposed to cytokines are metabolically less responsive to glucose stimulation and present signatures of oxidative stress

**Figure 2:**
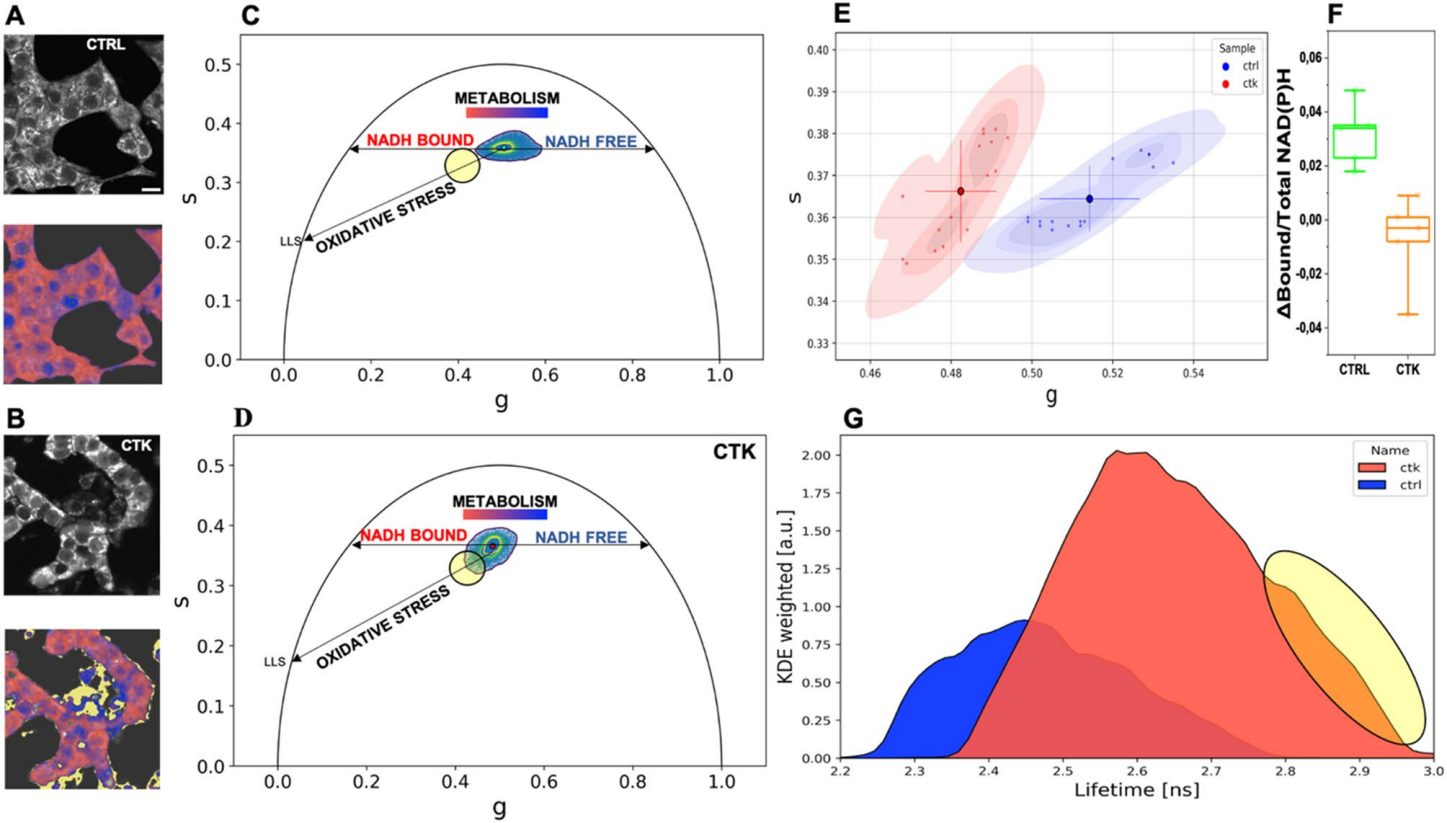
Phasor-FLIM analysis of cell autofluorescence. (**A**–**B**) Exemplary images of total NAD(P)H intensity of live INS-1E cell clusters. As shown on the bottom images, the color-bar defines the metabolic path from NAD(P)H in the bound state (red) to NAD(P)H in the free state (blue). (**B**) Pixels with LLS species are colored in yellow, according to the position of the yellow cursor in the phasor plot. LSS species are signatures of oxidative stress induced by cytokines. (**C-D**) Exemplary images and cumulative lifetime-based color map for NADH metabolic trajectories of control (**C**) and treated-samples (**D**). (**E**) Scatter plot of the average values of distinct phasor distributions, each relative to distinct acquired cells at the maintenance conditions. Blue squares represent control, while magenta circles show the cytokine-treated samples. Mean and SD were representative in the scatter plot (n = 16 frames; 3 independent experiments). A Mann–Whitney test was performed (****P < 0.0001). (**F**) Metabolic response for glucose stimulation (n = 5; 2 independent experiments) shows a NADH bound-shift in control sample, while for cytokine-treated samples is approximately zero. Data were presented as box plots with whiskers at the 5th and 95th percentiles, the central line at the 50th percentile, and the ends of the box at the 25th and 75th percentiles. A Kolmogorov–Smirnov test was performed (**P < 0.01). (**G**) Kernel density estimate of the lifetime probability distribution.

As mentioned above, FLIM on cell intrinsic signals can be exploited to extract quantitative information on both cell metabolic status and the emergence of oxidative-stress signatures^32–36^. To start, autofluorescence lifetime was measured in control cells and in cytokine-treated cells under maintenance culturing conditions (11-mM glucose concentration in RPMI medium) (Fig. 2A,B, upper panels in greyscale). Cumulative phasor plots of the lifetimes measured in control and cytokine-treated cells are reported in Fig. 2C,D: as expected based on previous results^32,33^, intracellular lifetimes are intrinsically multi-exponential, being the composition of multiple pure species, i.e. free NAD(P)H, enzyme-bound NAD(P)H and, eventually, oxidized lipid species in presence of oxidative stress. Two main characteristics of the phasor cluster are affected by cytokine treatment. First, the phasor-cluster barycenter changes position along the so-called “metabolic segment” upon exposure to cytokines, in particular moving towards an increase of the bound/free ratio of NAD(P)H forms (compare cumulative plots in Fig. 2C,D; see also the two exemplary images in Fig. 2A,B, lower panels, color-coded according to the metabolic trajectory). Cumulative results on barycenter position from the whole population of acquired cells (n = 16 cells from N = 3 independent experiments) are reported in Fig. 2E. Once assessed that cytokine-treated cells are ‘primed’ towards high bound/free NAD(P)H ratios, we probed the ability of cells in this condition to respond to glucose pulsed stimulation (see Materials and Methods for details on the protocol applied). Interestingly, as compared to control cells, cytokine-exposed ones showed a markedly reduced metabolic response to glucose in terms of phasor shift toward higher values of the bound/free NAD(P)H lifetime ratio (Fig. 2F). The second characteristic of the phasor cluster that is affected by cytokines is the overall shape of the cluster, presumably owing to the increased contribution of long-lifetime species (hereafter referred to as ‘LLS’, previously demonstrated to be the result of ROS production^36^). Indeed, the cumulative phasor from the whole cytokine-treated sample (n = 16; frames from 3 independent experiments) clearly shows an elongation towards longer lifetimes with respect to control (Fig. 2D), a result that is evident also by plotting the overall lifetime distribution (Fig. 2G). The ‘elongation’ of the lifetime distribution toward LLS upon cytokines exposure can be highlighted in the phasor plot using a cursor (black circle in Fig. 2C,D), which in turn identifies regions in the cells where LLS are mostly contributing to the measured lifetimes (yellow pixels in Fig. 2A,B, additional exemplary images are reported in Fig. S4).

### Pro-inflammatory-cytokine treatment is associated with altered morphological properties of mitochondria

**Figure 3:**
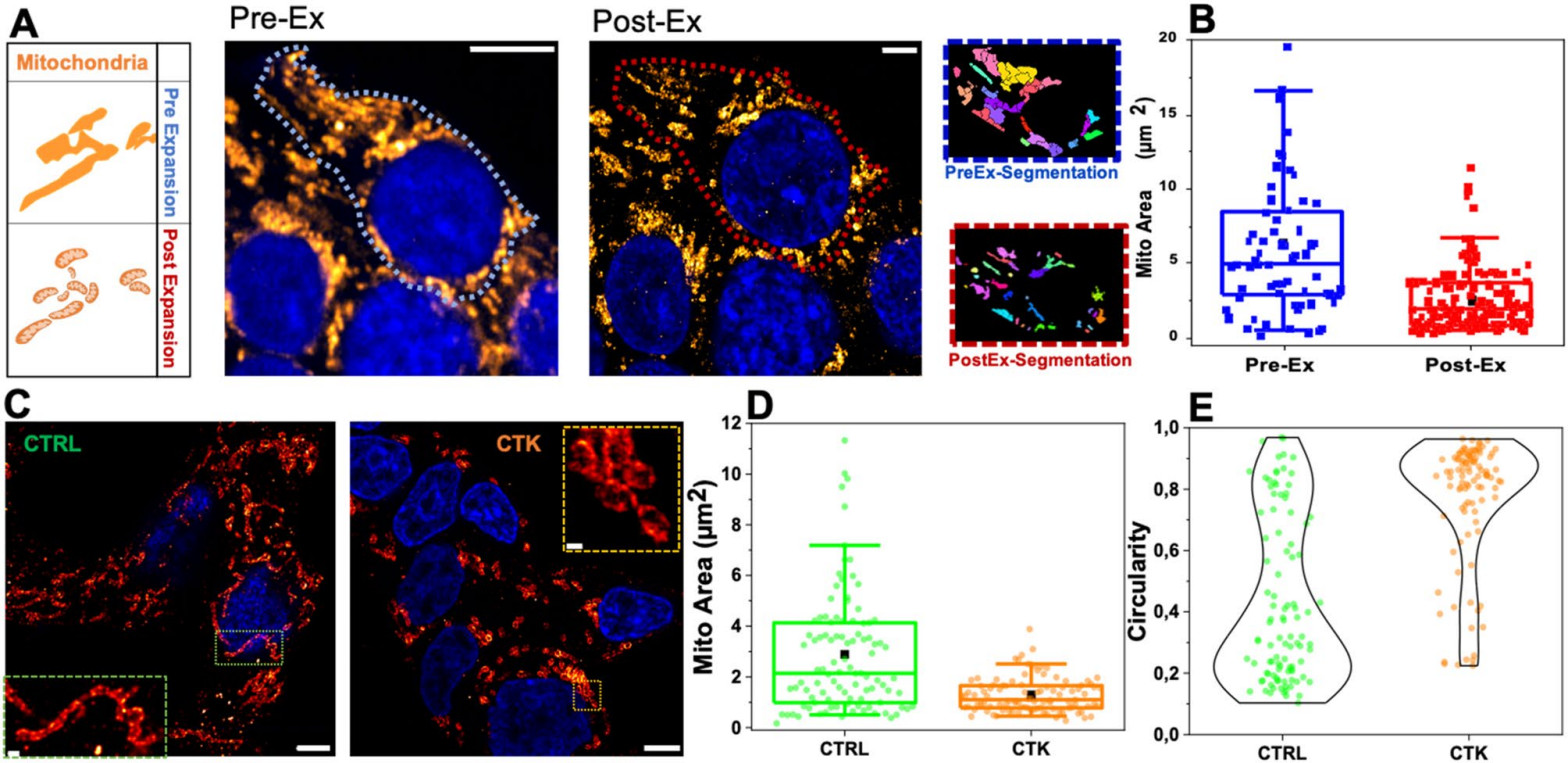
ExM for mitochondria investigation. (**A**) Mitochondrial network confocal images and algorithm segmentation in unexpanded and expanded INS1E cells stained for TOM20. (**B**) Box plot shows morphometric analysis performed by using the MorphoLibJ plugin. The mitochondrial area is expressed as µm^2^. Data are presented as box plots with whiskers at the 5th and 95th percentiles, the central line at the 50th percentile, and the ends of the box at the 25th and 75th percentiles (n. of mitochondria = 100 for control and treated samples; 2 independent experiments). A Mann–Whitney test was performed (***P < 0.001, ****P < 0.0001). Cells were acquired by confocal microscope using 405 and 488 excitation light, with 63x/NA1.4 objective lens. Scale bar 10 µm. (**C**) Representative images of expanded INS-1E cells stained for TOM20 after incubation with fresh medium (CTRL) and cytokines (CTK) for 24 h. Scale bar 10 µm. The structural analysis on expanded mitochondria shows a reduced area (**D**) and high circulatory value (tending to 1) (**E**) in cytokine-treated samples.

A number of studies place mitochondrial dysfunction as an hallmark of Diabetes pathogenesis^17,18,21^. As shown in Fig. 3A, mitochondria form a highly interconnected and dense tubular network in INS-1E cells, making it difficult to investigate their number and structural features using a conventional microscope. Therefore, after 24 h of cytokine treatment, cells were fixed, immunostained by TOM20, a component of the translocase of the outer membrane (TOM) complex, and, finally, expanded. ExM allowed accurate segmentation and quantification of the number and structure of such subcellular organelles. As expected, expanded TOM20-labeled mitochondria were smaller in size (area expressed as µm^2^; Fig. 3B) and greater in number (Fig. S5A) per cell after rescaling for the EF. Also, we observed a reduced cytoplasmic area (Fig. S5B), indicating better individually resolved mitochondria. Then, cytokine-treated cells were compared to the control ones for mitochondria morphometric investigation. As shown in Fig. 3C, the classical filamentous pattern of labeled mitochondria appears fragmented into numerous small punctate particles upon exposure to cytokines. This evidence mirrors quantitatively into the analysis of mitochondrial area and circularity parameters (Fig. 3D,E): the former changes from 2.88 ± 0.29 µm^2^ in control cells to 1.29 ± 0.07 µm^2^ in treated cells (reduction of 55%), the latter from 0.43 ± 0.03 in control cells to 0.77 ± 0.02 in treated cells. Overall, mitochondria in treated cells appear smaller in size and altered in shape, structural features typically associated with dysfunctional mitochondria^42^ and, presumably, metabolic stress.

### Pro-inflammatory-cytokine treatment is associated with ISGs decreased number, altered localization and dynamics

**Figure 4:**
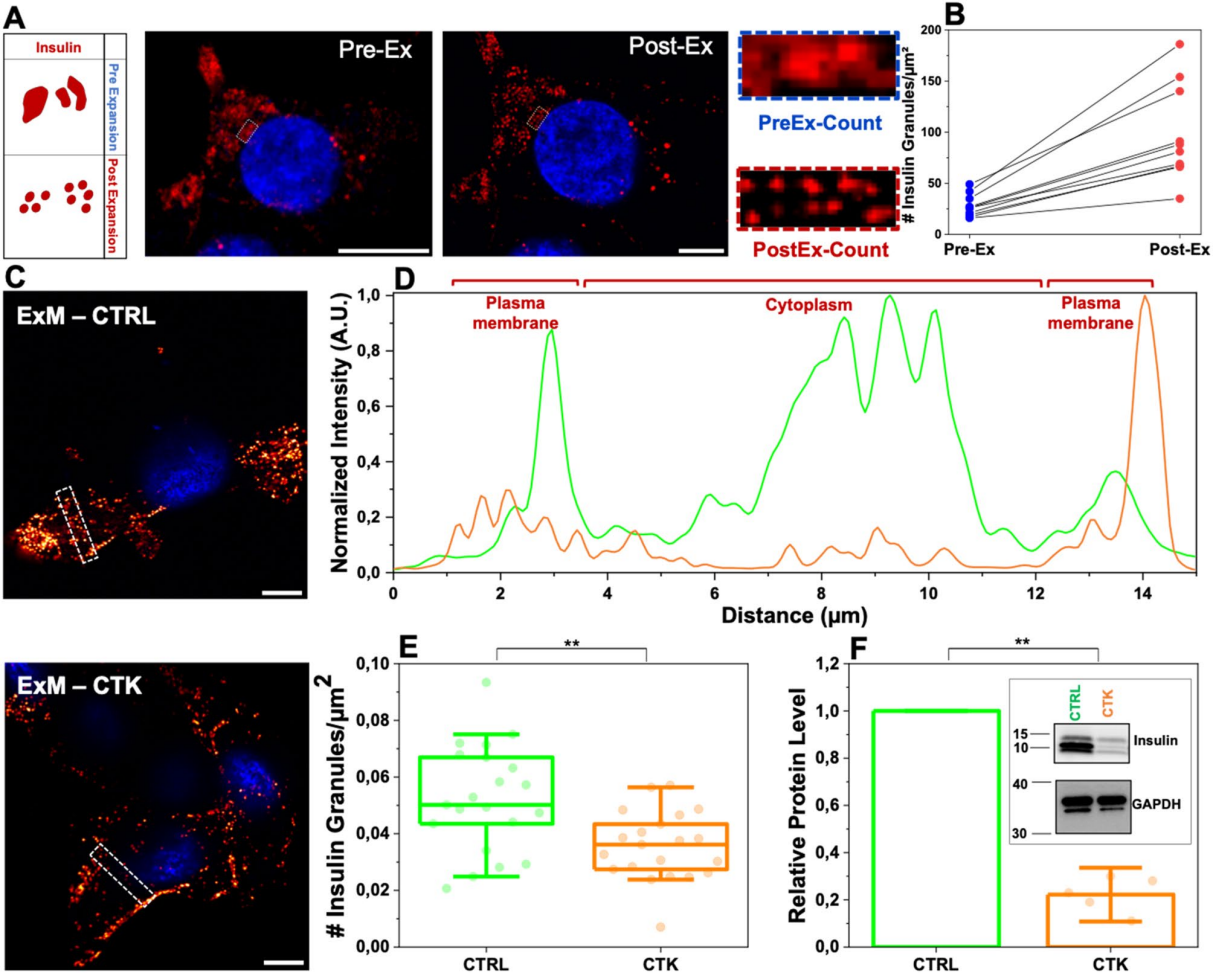
Proinflammatory cytokines induce secretion of ISGs. (**A**) Pre- and post-expanded INS-1E cells labeled for insulin. The inset shows a zoomed-in region highlighting the improved resolution of expanded samples. (**B**) ISG counts before versus after expansion for 25 different cells (each circle represents the single cell; n = 3 independent experiments). (**C**) Control and cytokine-treated sample of INS-1E cells stained for ISGs in maintenance condition. Cells were acquired by confocal microscope using 405 and 488 excitation light, with 63x/NA1.4 objective lens. Scale bar 10 µm. (**D**) Profiles of intensity taken along the 2 × 15 µm^2^ white square. The plot profiles show a more homogeneous distribution of IGs in the cytoplasm in the control (green) with respect to cytokine-treated samples (red). (**E**) Granule count in CTRL and CTK expanded samples (ExM), showing a statistically significant reduction of the insulin content in CTK samples. Data were presented as box plots with whiskers at the 5th and 95th percentiles, the central line at the 50th percentile, and the ends of the box at the 25th and 75th percentiles (n = 21 cells; 3 independent experiment; the total number of optical sections examined was 337 for CTRL samples and 344 for CTK samples). (**F**) Total proteins were extracted, and the expression of insulin were assessed by Western blotting (WB). GAPDH was used as a control for protein loading. Protein signals were quantified and corrected for the corresponding GAPDH value and expressed as fold change compared to untreated cells (CTRL) (n = 5 independent experiments). (**p ≤ 0.01; Non-parametric Mann Whitney test; significantly different from the control condition at 24 h of incubation).

As reported in Fig. 4A,B, the de-crowding effect imposed by ExM favored identification of single granules and, as a consequence, their proper counting (e.g. the number of ISGs detected markedly increases in post-expansion specimens with respect to pre-expansion ones; n = 25 cells, 3 independent samples). Worthy of mention, post-expansion ISGs reveal a donut-shaped staining pattern, which is not detectable in pre-expansion ones (exemplary image in Fig. S6): the hole of the donut might correspond to the crystalline inner core of the granule, eventually not reached by primary and secondary antibodies due to steric hindrance. Already at a first visual inspection (exemplary images are shown in Fig. 4C), it appears clear that ISGs are more uniformly distributed in control cells as compared to cytokine-treated ones, in the latter being preferentially accumulated at the cell border (i.e. close to the plasma membrane). To better highlight this difference, Fig. 4D reports on the average fluorescence intensity recorded in a ROI drawn across the cytoplasm and borders of the cell (dashed rectangular boxes in Fig. 4C). As expected, in a control cell ISG-specific signal is present almost throughout the cell environment (green trace in Fig. 4D), while in cytokine-treated cells it is markedly higher at the borders as compared to the cytoplasm (orange trace in Fig. 4D). To accurately estimate the ISG number, the entire cellular volume was probed in expanded specimens by means of z-stack acquisitions with a z-stack step of 1 µm. Of note, a ~ 40% reduction of the ISGs density (i.e. ISG number/µm^2^) was observed in cytokine-exposed cells as compared to control cells (Fig. 4E). Such reduction in ISG content upon exposure to cytokines was confirmed by independent Western-Blot (WB) analysis of the total insulin content in cell lysates (Fig. 4F, see also Fig. S7 for uncropped WB) and supports the current model of aberrant insulin secretion induced by cytokines^7–10^. Please note that in the specific case of ISGs, the ExM-derived information on ISG density and localization was complemented with dynamic information on the population of residual ISGs labelled using ZIGIR (a fluorescent granule indicator with Zn^2+^-chelating properties^43^; see Materials and Methods) (Fig. S8A) and analyzed by spatiotemporal image correlation spectroscopy in the form of *i*MSD (i.e. imaging-derived Mean Square Displacement^44,45^) (Fig. S8B-C). Of note, the ISG dynamic properties showed statistically significant alterations (Fig. S8D-E): in particular, the α coefficient decreased under cytokine treatment while, concomitantly, D_m_ increased. These data suggest that the residual granule population after 24-h exposure to cytokines is less prone to perform active transport as compared to granules from untreated cells.

### Super resolution via expansion microscopy highlights structural alteration of microtubules in cytokine-exposed INS-1E cells

**Figure 5:**
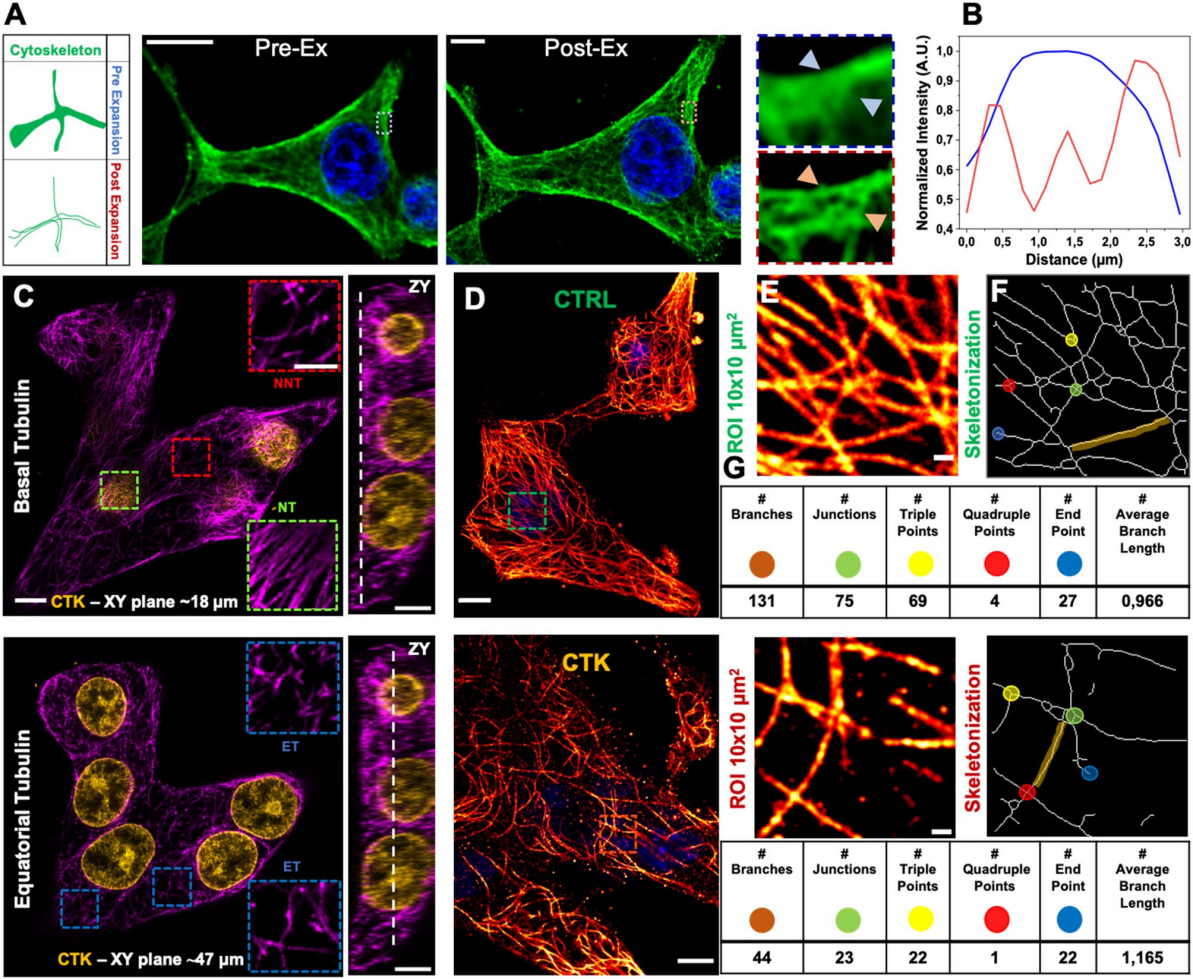
Proinflammatory cytokines promote microtubule rearrangement in INS-1E cells. (**A**) Pre- and post-expansion confocal images of INS-1E stained for α-tubulin, with magnified views of boxed regions and (**B**) profiles of intensity taken along the blue and red arrows. (**C**) Representative Z-stack image of treated sample and ROI collection at different planes. ROI were taken at the basal plane (Z-plane ~ 18 µm)—in the proximity of the nucleus (termed Nuclear Tubulin; NT) and outside the nucleus (termed No Nuclear Tubulin, NNT)—and at the equatorial level (Equatorial Tubulin, ET; Z-plane ~ 47 µm). The ZY projection shows an example of the localization along the Z-axis. Scale bar 10 µm. (**D**) Representative images and microtubule-network analysis of expanded INS-1E cells untreated (control, CTRL) and treated with cytokines (CTK). Cells were stained for α-tubulin and DAPI and acquired by confocal microscope using 405 and 488 excitation light, respectively, with 63x/NA1.4 objective lens. (**E**) ROI of 10 × 10 µm^2^ were then analyzed by FiNTA and skeletonized through Fiji (**F**), to quantify the reduction in the number of branches (# Branches), junctions (# Junctions), triple and quadruple points (# Triple and Quadruple points), endpoints (# Endpoints), and average bench length (**G**). Scale bar 10 µm.

**Figure 6:**
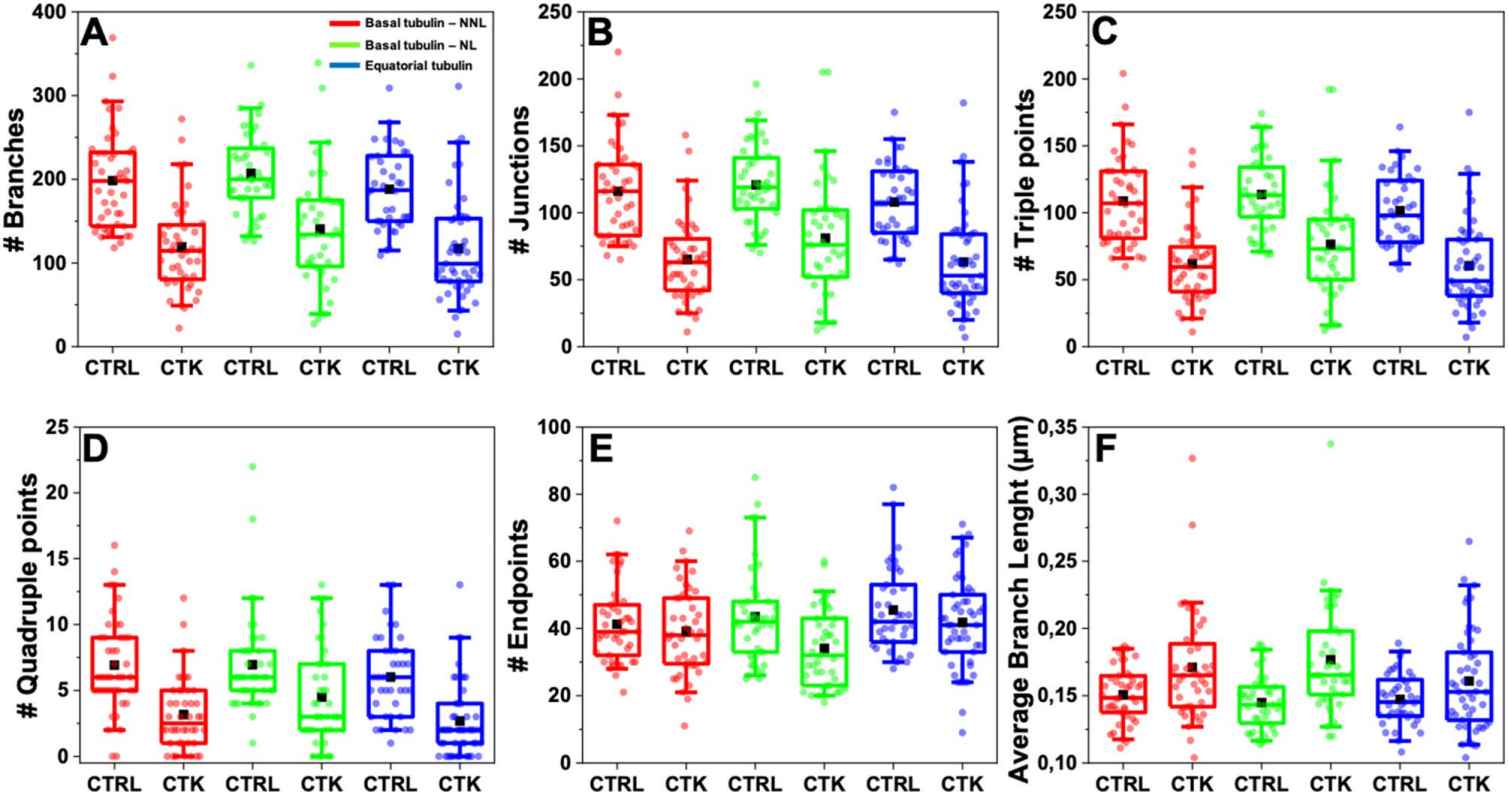
Quantification of microtubule rearrangement in INS-1E cells after cytokine treatment in different areas of the cell. Quantification of MT density per ROI: # Branches (**A**), # Junctions (**B**), # Triple (**C**) and Quadruple (**D**) points, # Endpoints (**E**), and Average Branch Length (**F**). Cytokine-treated specimens were significantly different from the control condition in each plane. In addition, the average branch length increases in cytokine treated samples with respect to the control. Data were presented as box plots with whiskers at the 5th and 95th percentiles, the central line at the 50th percentile, and the ends of the box at the 25th and 75th percentiles (n ≥ 39; 3 independent experiment). A Mann–Whitney test was performed.

**Figure 7:**
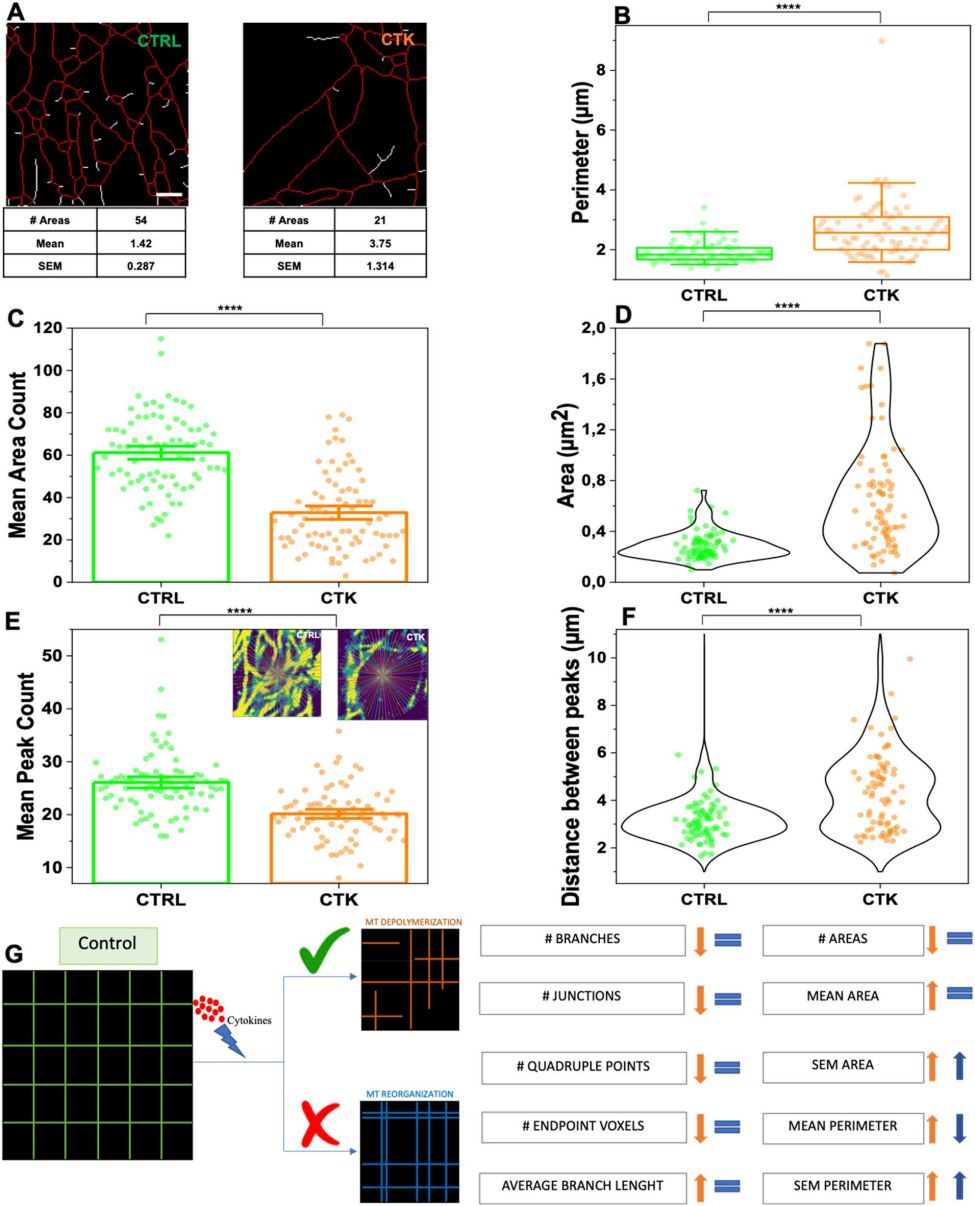
Proinflammatory cytokines promote microtubule rearrangement in INS-1E cells. (**A**) MT opening analysis by using MorphoLibJ algorithm. The number of opening and mean areas were calculated for each ROI in control and treated samples. (**B**) Treatment with cytokine induces significant increase in corollas size, including mean perimeter. Data were presented as box plots with whiskers at the 5th and 95th percentiles, the central line at the 50th percentile, and the ends of the box at the 25th and 75th percentiles (n = 78; 3 independent experiments). Bar ± SEM (**C**) and Violin plot (**D**) show the number of MT openings and mean area, respectively, for each ROI. (n = 76; 3 independent experiments). Bar ± SEM (**E**) and violin plot (**F**) show the mean peak count and mean distance between peaks per ROI based on a intensity distribution analysis of 175 lines passing from the frame center in control and cytokine-treated samples (boxes in **E**). A Mann–Whitney test was performed. Scale bar 10 µm. (**G**) Schematic model of tubulin alterations after cytokine treatment. Data on the left were calculated manually, data on the right were calculated with MorphoLibJ. These results demonstrate that the orange model is more affine to the ExM data.

To gain insight into the effect of pro-inflammatory cytokines on the structural organization of microtubules in INS-1E cells, we combined immunofluorescence, which provides molecular specificity, with ExM, which physically magnifies the sample and allows super-resolved images in conventional diffraction-limited microscopes. We started by examining the effect of cytokines on microtubules. In detail, cells were exposed for 24 h to cytokines (IL-1β 10 U/ml and IFN-γ 100 U/ml diluted in RPMI) in the maintenance medium (see Materials and Methods), then fixed, labeled with anti-α-tubulin antibody, and expanded using ExM protocol. As reported in Fig. 5A,B, ExM allowed to resolve individual microtubules that could not be distinguished with standard confocal microscopy (see profiles of intensity in Fig. 5B). To analyze the impact of cytokines on MT organization, we selected three different 10 × 10 µm^2^ Regions Of Interest (ROIs) within a complete Z-stack acquisition of each cell. These ROIs were: *i*) basal tubulin – No Nuclear Localization (NNL) (α-tubulin found in contact with the glass and in the first planes of the Z-stack), *ii*) basal tubulin – Nuclear Tubulin (NT) (α-tubulin found in proximity of the glass and beneath nuclei), and *iii*) equatorial tubulin (ET), both in normal and cytokine-treated samples (shown in Fig. 5C for a representative cytokine-treated INS-1E cell). For each cell, we extracted more than one ROI from the basal and equatorial cytoplasm to ensure that we covered the entire cell surface and obtained an optimal signal-to-noise ratio for efficient tracing (Fig. 5C). The expanded images in Fig. 5D for INS-1E cells labeled for α-tubulin show a significant decrease in MT density in cells treated with cytokines (bottom panel) compared to control cells (upper panel). To quantify these changes, we used the FiNTA algorithm to trace the cytoskeletal network in 10 × 10 µm^2^ ROIs (Fig. 5E)^46^. This generated a binary mask of the network (Fig. 5F) that we further processed resorting to the Skeletonize function^47^ to extract information about an ensemble of parameters that describe the structure of the cytoskeleton, including the number of branches (portion of segments connecting by end-points, or endpoints – junctions, or junctions – junctions; brown in Fig. 5F), junctions (green), triple points (yellow) and quadruple points (red) (junctions with exactly 3 and 4 branches, respectively), endpoints (in blue), as well as the average lengths of branches (Fig. 5G). Exposure to cytokines significantly reduced the number of branches (control: 198.0 ± 4.7, cytokines: 125.0 ± 5.3; reduction of 37%) (Fig. 6A), junctions (control: 115.1 ± 2.8, cytokines: 69.3 ± 3.3; reduction of 40%) (Fig. 6B), triple points (control: 108.1 ± 2.7, cytokines: 65.8 ± 3.0; reduction of 39%) (Fig. 6C), quadruple points (control: 6.6 ± 0.3, cytokines: 3.4 ± 0.3; reduction of 48%) (Fig. 6D), and endpoints (control: 43.3 ± 1.2, cytokines: 38.6 ± 1.1; reduction of 11%) (Fig. 6E). Furthermore, we observed a significant increase in the average branch length (control: 0.330 ± 0.004 µm, cytokines: 0.380 ± 0.007 µm; reduction of control 13%) (Fig. 6F). Finally, binary images were processed with MorphoLibJ (Fiji plugin, see Methods) to segment the MT network and calculate the number, area (Fig. 7A) and perimeter of MT meshes (Fig. 7B). This analysis revealed that cytokine-treated samples had a decrease in mean area number (Fig. 7C) and a twofold increase in the MT opening size (0.487 ± 0.010 µm^2^ in treated samples compared to 0.260 ± 0.004 µm^2^ in control ones) (Fig. 7D). This structural outcome was also confirmed by the intensity distribution generated through the repetition of 175 lines running across the center of the frame (Fig. 7E and Fig. S9). Statistical analysis of the number and position of peaks along these lines indicated that cytokines-treated samples had a lower number of MT filaments compared to control samples (Fig. 7E), with a greater distance between peaks (Fig. 7F). WB analysis of α-tubulin shows that cytokine treatment does not reduce the tubulin content in INS-1E cells (Fig. S7 and Fig. S10A). Such a MT remodeling can be explained with depolymerization and/or spatial re-organization of the cytoskeletal mesh. By modeling the cytokine-induced MT alteration using a basic network (Fig. 7G), we confirmed that the MT destabilization can be interpreted as a depolymerization process. At this point we completed the analysis by performing the same experiments on cortical actin. Phalloidin, typically used to label cortical actin in cells, lacks amine groups for gel anchoring. To tackle this issue and retain the fluorescence signal after expansion, in the first step fluorophore-conjugated Phalloidin was labeled using anti-fluorophore antibodies, followed by hydrogel synthesis, enzymatic digestion, and expansion (see Materials and Methods for more details). After confocal acquisition, the images were processed in order to calculate filament length, number of branches, junctions, triple and quadruple points, endpoints, shape and size of actin corrals. Since cortical actin shows a diverse and heterogeneous arrangement in INS-1E cells (Fig. S11A), the entire cell was analyzed and obtained results normalized by the cell area (and by the EF). Data analysis yielded no significant modifications of the selected actin structural parameters (results are reported in Fig. S11B—E). These results from imaging are accompanied by WB analysis which detected no variation in the total amount of actin (Fig. S7 and S10B). Although the nanoscale intracellular organization of the actin meshwork is not affected by cytokines, a substantial increase in the number of micrometric cytoplasmic protrusions was observed in treated cells as compared to control ones (~ 72% increase, Fig. S11F-G). Such structures, also known as cytoplasmic “blebs”, are a special type of cell protrusions that are driven by intracellular pressure or caused by oxidative-stress^48^, and typically enriched in actin filaments^49^.

## Discussion

In this work, we exploit a combination of label-dependent and label-free high-resolution optical-microscopy tools for the direct imaging of the intracellular environment in a homogenous population of INS-1E β-cells exposed to IL-1β and IFN-γ pro-inflammatory cytokines for 24 h. Preliminarily we verified that, in our experimental system, cytokine treatment is associated with a clear shift of β-cell metabolic status towards higher bound/free NAD(P)H lifetime ratios, a response that recalls what previously measured under glucose-stimulation conditions^32^. The increase of the ratio between protein-bound and free NAD(P)H species is classically interpreted as an increase in the oxidative-phosphorylation activity of the cell^32^, a step required in turn to rapidly increase the ATP/ADP ratio and stimulate the cascade of biochemical reactions leading to insulin secretion. In this view, the similarity between β-cell response to cytokine- and that to glucose-exposure measured by FLIM is not surprising, as both stimuli are known to promote insulin secretion. Along the same reasoning, cytokine-induced β-cell shift towards higher bound/free NAD(P)H lifetime ratios, besides being a putative signature of insulin secretion, also sets a condition that inevitably makes the β cell less responsive to further glucose stimulation, as confirmed here and observed previously exposing INS-1E cells to hyperglycemia^32^. At this stage, it is worth noting that variations of the bound/free NADPH balance can also reflect variations in the identity and/or expression-level of enzymes involved in NADPH processing. Indeed, we recently demonstrated that non-secretory cells show a different response to glucose stimulation as compared to INS-1E cells, presumably in light of the different enzymatic array expressed^32^. Of interest for the present case, it was demonstrated that prolonged exposure of human islets, mouse islets, and clonal β-cell lines to cytokines is associated with a significant increase in the expression of several NADPH oxidases, then implicated in the generation of ROS, in mitochondrial dysfunction and, eventually, β-cell demise (for a detailed review see Ref.^12^). In keeping with this picture based on standard “omics” approaches^50–52^, here label-free infrared microscopy highlights the signature of ROS-generated lipid-oxidation products. In such a metabolically-impaired cellular landscape, ExM-based super-resolution imaging unveils marked cytokine-induced structural fragmentation of the microtubule mesh, compatible with a de-polymerization process. Regard to this latter, it is worth noting that recent findings indicate that microtubule depolymerization increases the capacity of β cells for glucose-stimulated insulin secretion (GSIS)^53^, in partial contradiction with previous reports (refs^25,54–56^). In particular, Trogden and co-workers, by an imaging-based analysis of the spatiotemporal patterns of secretion events, showed that microtubule depolymerization activates otherwise dormant β-cells via initiation of secretion clusters (hot spots) and, also, enhances overall secretion by introducing both additional clusters and scattered events^53^. Interestingly, in presence of extensive microtubule depolymerization induced by Nocodazole, the timing of clustered secretion is dysregulated, extending the first phase of GSIS and causing over-secretion^53^. Interestingly, the similarities between the effects induced by cytokines and those induced by glucose stimulation extend to what observed by ExM and spatiotemporal correlation spectroscopy on ISGs. In fact, by imaging on fixed cells at 50-nm spatial resolution and *i*MSD-based analysis of time-lapse acquisitions on live cells, we observed that, after 24-h exposure to cytokines, the surviving population of ISGs is substantially decreased in number (~ 40% less), mis-localized (closer to the cell border), and with altered dynamics (increased local diffusivity and decreased propensity to perform active transport). Regard to this latter observation, it is worth noting that a similar change in the ISG dynamic properties was already reported by some of us upon overexpression of the ISG-specific transmembrane protein Phogrin or in conditions of cholesterol overload^45^, which both are stimuli associated with decreased cell responsiveness to glucose stimulation, similarly to what produced here using pro-inflammatory cytokines. If the dynamic properties of the surviving ISG population speak of a reduced commitment of granules for secretion, the observed decrease in their number seems to confirm the ability of cytokines to induce aberrant insulin secretion observed by others on human-derived islets by a combination of secretion assays, RNA sequencing, and mass spectrometry^10^. The emerging scenario, based on our data, is compatible with pro-inflammatory cytokines acting at first as stimulators of aberrant insulin secretion, a massive event then contributing to the reduced β-cell sensitivity to further glucose stimulation, in keeping as well with previous reports^10,11^. In general, benchmarking by direct imaging the intracellular landscape of β-cells exposed to pro-inflammatory cytokines holds the potential to set the guidelines for data acquisition/interpretation in future studies. Also, present results provide a reference with which to compare the effect of any other stimuli, even protective ones (e.g. cytoprotection against oxidative stress and mitochondrial dysfunction^57^). Interesting development directions certainly include the completion of the intracellular targets of investigation in clonal β-cell lines and studying β cells in their natural context, i.e. the intact Langerhans islet from *ex-vivo* fresh samples or paraffin-embedded tissues, potentially derived also from donors naturally exposed to cytokines (e.g. T1D donors). Along this path, we envision a crucial role of machine-learning-based strategies aimed at facilitating cell type recognition and, eventually cell functional and/or structural changes^58^.

## Materials and methods

### Cell culture

INS-1E cells (kindly provided by Prof. C. Wollheim, University of Geneva, Medical Center) were maintained in culture at 37 °C, 5% CO_2_ in RPMI 1640 medium containing 11.1 mmol/L D-glucose, supplemented with 10% heat-inactivated fetal bovine serum (FBS), 10 mmol/L HEPES, 2 mmol/L L-Glutamine, 100 U/mL penicillin–streptomycin, 1 mmol/L sodium-pyruvate, 50 μmol/L tissue culture grade β-mercaptoethanol. For ExM experiments, cells were plated at 70% confluency on 18 mm coverglass and grown for 48 h. For lifetime experiments, cells were plated at 70% confluency onto sterilized and fluorescence‐microscopy‐suitable dishes (IbiTreat µ‐Dish 35‐mm, Ibidi) for 24–48 h. Then, cells were exposed to cytokines (IL-1β 10 U/ml and IFN-γ 100 U/ml diluted in the complete medium) for 24 h. For control samples, cells were washed and replaced with a fresh complete medium. Cells used in this study resulted negative for the presence of mycoplasma contamination.

### Western blot

Western blot technique was used to quantify and compare the intracellular concentration of proteins in cytokine-treated INS1-E cells and in the untreated control. INS-1E cells were treated with Cytokines (IL-1β 10 U/ml IFN-γ 100 U/ml diluted in 1 ml of medium RPMI) for 24 h. For western blotting, the cells were lysed in 95 °C Laemmli buffer (60-mM Tris–HCl, pH 6.8, 2% SDS, 10% glycerol). Protein concentration was measured using Pierce™ BCA Protein Assay Kit (Thermo Fisher) following manufacturer's instructions. Samples were then prepared by adding DTT (dithiothreitol) to a final concentration of 100 mM and bromophenol blue to the appropriate volume of cell lysate in order to load 20 ug of total lysate for each sample. Samples were subsequently subjected to SDS-PAGE and transferred onto a nitrocellulose membrane. Non-specific protein binding was blocked by incubation with 5% non-fat dry milk in PBST (phosphate buffer saline with 0,05% Tween-20) for 1 h. Primary antibodies were incubated overnight at 4 °C in PBST with 5% milk with gentle shaking. After three washes with PBST, HRP-conjugated secondary antibodies were incubated for 1 h at room temperature. Complete list of employed primary and secondary antibodies is reported in Table S1. After washing, membranes were incubated with ECL™ Prime Western Blotting System (GE Healthcare) and the chemiluminescence signal was detected using ChemiDoc MP Imaging System (BIORAD). Reported values from independent experiments are normalized on the GAPDH signal and presented as a percentage of the control sample (not treated).

### Live-cell imaging

For phasor-FLIM metabolic experiments, living INS-1E cells were examined after 24 h of cytokine exposure in the maintenance condition (RPMI 1640 medium containing 11.1 mmol/L D-glucose, supplemented with 10% heat-inactivated FBS, 10 mmol/L HEPES, 2 mmol/L L-Glutamine, 100 U/mL penicillin–streptomycin, 1 mmol/L sodium-pyruvate, 50 μmol/L β-mercaptoethanol) at 37 °C. For the glucose stimulation experiments, cells (control and cytokine-treated samples) were washed with SAB (114 mM NaCl, 4.7 mM KCl, 1.2 mM KH2PO4, 2.5 mM CaCl2, 1.16 mM MgSO4, and 20 mM HEPES (pH 7.4)) supplemented with 2.2 mM glucose, and then incubated with SAB 2.2 mM glucose for 45 min (low glucose concentration) at 37 °C, 5% CO_2_. Then, glucose was added to reach a final concentration of 16.7 mM (high glucose concentration). The same cell clusters were acquired with 2-photon microscopy at low and high glucose concentrations. For iMSD experiments, INS-1E cells were treated with Cytokines (IL-1β 10 U/ml IFN-γ 100 U/ml diluted in 1 ml of medium RPMI) to investigate the diffusive motion of insulin granules after 24 h of administration. Insulin granules were marked by ZIGIR, a fluorescent probe that labels Zinc-rich granules^43^. INS-1E cells were plated into 1 ml IBIDI plates (81,156: μ-Dish 35 mm, high ibiTreat: Ø 35 mm, high wall) incubated at 37 °C and 5% CO_2_ for 24 h. For the labeling process, cells were incubated 1 μM ZIGIR into the cells’ medium and incubated for 15 min.

### Cell fixation

Control and cytokine-treated cells were fixed according to the structure to stain. For tubulin, the specimens were incubated in the pre-extraction buffer consisting of 0.1 mM Pipes, 1 mM MgCl_2_, 1 mM EGTA, 0.5% Triton, and 3% paraformaldehyde (PFA) in dH_2_O for 1 min at room temperature (RT) and then fixed with 3% PFA + 0.1% glutaraldehyde (GA) for 10 min at RT, following three washes in 1 × phosphate buffer saline (PBS), 5 min each. For the actin staining, the samples were fixed with 4% PFA in PBS for 30 min at RT and washed 3 times with PBS, 5 min each. For mitochondria, cells were fixed with 3% PFA + 0.1% GA in PBS for 30 min at RT and washed 3 times in PBS, 5 min each. Finally, for the insulin granule staining, cells were fixed with 4% PFA in PBS for 30 min at RT and washed 3 times with PBS, 5 min each.

### Immunostaining and functionalization

After fixation, cells were permeabilized with PBS + 0.1% triton X-100 (PBST) for 10 min at RT, washed 2 times with PBS, and then blocked with 2% bovine serum albumin (BSA) for 30–45 min at RT. The samples were incubated with the following primary antibodies: mouse anti-α-Tubulin (diluted 1:200 in PBST) for 2 h at RT; rabbit anti-Insulin and rabbit anti-TOM20 (diluted 1:200 and 1:100, respectively, in PBS + 0.1% Tween) overnight at 4 °C. then, after 3 washes with PBST (for tubulin) or PBS + 0.1% tween (for mitochondria and insulin) for 10 min each, the specimens were incubated with secondary antibodies anti-Rabbit Alexa Fluor 488 and anti-mouse Alexa Fluor 488, diluted 1:200 in PBST and PBS + 0.1% tween, respectively (see Table S1). For Actin, we adapted the protocol developed by Park et al^59^. Shortly, cells were stained with Alexa Fluor 488-conjugated phalloidin (diluted 1:5 in PBST) for 1 h at RT. After washing three times in PBST, cells were stained for 60 min with an anti-fluorophore antibody (rabbit anti-Alexa Fluor 488 antibody, diluted 1:100 in PBST) for 1 h at RT. Next, cells were washed three times with PBST. Cells were then stained for 2 h with a secondary antibody (Anti-rabbit Alexa fluor 488, diluted 1:100 in PBST) (see Table S1). The stained samples for tubulin, actin, insulin, and mitochondria were then washed 3 times with PBS, 10 min each, and then functionalized with 0.1 mg/mL of 6-((acryloyl)amino)hexanoic Acid, Succinimidyl Ester (AcX, ThermoFisher, A20770) for ≥ 6 h at RT. After the functionalization step, the samples were washed 3 times with PBS, 10 min each.

### Polymerization, digestion, and expansion

The stained and functionalized specimens were soaked in a gelled solution consisting of 8.625% sodium acrylate, 2.5% acrylamide, 0.15% N,N′-methylenebis(acrylamide), 2 M NaCl, 1 × PBS, diH2O, 0.2% ammonium persulfate (APS) and 0.2% (v/v) tetramethylethylenediamine (TEMED), with TEMED and APS last added in this order. The gelation solution (~ 50 μL) is placed on the hydrophobic surface, and the coverglass is placed on top of the solution with cells facing down. Gelation is allowed to proceed at RT for 1–1.30 h. After gelation, the gels attached to the coverglass were removed and placed in a digestion buffer consisting of 50 mM Tris–HCl (pH 8), 1 mM EDTA, 0.5% Triton X-100, 1 M NaCl supplemented with 8 units mL^−1^ proteinase K added freshly. Gels were digested overnight at RT with gentle shaking and then washed with 1 μg/mL DAPI in PBS for 10 min. Then the hydrogel is moved into a 60 mm petri dish and soaked in ~ 50 mL DI water to expand it. Water is exchanged every 30 min and 4 times until expansion is complete. At the final expansion, small gel pieces were cut from the gelled samples and observed by confocal microscopy.

### Fluorescence microscopy

Unexpanded and expanded fixed samples stained for tubulin, actin, insulin, and mitochondria were acquired with an inverted Zeiss LSM 800 confocal microscope (Jena, Germany). The acquisition was performed by illuminating the sample with a 405, 488, and 561 nm laser using a 63 × /NA 1.4 oil-immersion objective. DAPI, AlexaFluor 488, and AlexaFluor 568 fluorescence were collected between 410–510 nm, 510–590 nm, and 590–700 nm, respectively, with GaAsP detectors. Z-stacks with step sizes of 1 µm and 0.6 µm were acquired for insulin and tubulin staining, respectively, while tiled images were stitched into a mosaic (~ 895 µm × 895 µm) to find the same cell cluster in the unexpanded and expanded specimens. Stacks of images for iMSD experiments were performed with an inverted Zeiss LSM 800 confocal microscope (Jena, Germany) exciting the ZIGIR at 561 nm and collecting the fluorescence between 590 and 700 nm. The pinhole aperture was set at 1 Airy (53 μm) and a 63x/1.4 oil objective was used. 500 frames per image were collected and each frame was 256 × 256 pixels size, with acquisition time of 204.80 ms. Metabolic imaging was performed by an Olympus FVMPE-RS microscope coupled with a two-photon Ti:sapphire laser with 80-MHz repetition rate (MaiTai HP, SpectraPhysics) and FLIMbox system for lifetime acquisition (ISS, Urbana Champaign). NADH was excited at 740 nm and emission was collected using a 30 × /NA 1.0 planApo silicon immersion objective in the 420–460 nm range. Flimbox system calibration was performed by measuring the characterized mono-exponential lifetime decay of Fluorescein at pH = 11.0 (4.0 ns upon excitation at 740 nm, collection range: 480–570 nm). To prepare the calibration sample, a stock of 100 mmol/L Fluorescein solution in EtOH was prepared and diluted in NaOH at pH 11.0 for each calibration measurement. A 512 × 512 pixels image of FLIM data was collected into 25–30 frames, with an acquisition time typically of 1–2 min.

### Data analysis

For the expansion factor (EF) characterization, cells labeled for actin (for the cellular EF, n = 15) and mitochondria (for subcellular EF, n = 15) were acquired before and after expansion with confocal microscopy and calculated the EF by the ratio of cell and mitochondria area. The degree of sample deformation caused by ExM was calculated by comparing the pre- and post-Ex images stained for tubulin, actin, mitochondria and insulin (n = 3 for each labeled structures) using the structural similarity index (SSIM index) plugin. For the count of insulin granules, Z-stack acquisitions with low downsampling along the Z-axis were performed to image different granules in each plane (Z step = 1 µm). Then, Z-stacks were converted into a binary image by defining an appropriate threshold and analyzed with the “Analyze Particle” tool. The number of granules for a single cell was normalized for the cell area and then the percentage reduction of granules after cytokine treatment was calculated. For α-tubulin, Regions Of Interest (ROIs) of 10 × 10 µm^2^ at different planes along the Z-axis (basal and equatorial, see Results; n = 26, from 3 independent samples) were selected and processed by Fiji to improve the signal-to-noise ratio (SNR) (Process>Math>Subtract Background). Then, the tracing process was performed by FiNTA, to generate binary images of the MT meshwork. Finally, Finta-processed images were converted by the Fiji Skeletonize plugin (Plugin>Skeleton>Skeletonize (2D/3D)) into a binary image and then analyze using “Analyze Skeleton”, to achieve information on the number of branches, junctions, triple points, quadruple points, endpoint, and average branch length. For the calculation of the MT opening (number and average area), the binary images were analyzed by MorphoLibJ plugin (Plugin>MorphoLibJ>Segmentation>Morphological Segmentation>Border Image). To provide a way of characterizing the intensity distribution of the MT network in terms of branches’ average distance and number of intensity peaks, an adaptation of Garlick’s method^60^ was implemented in Python 3.6. Such features were calculated through an image- processing algorithm plotting intensity values along 175 diameters running across the circle inscribed in every square image. Consequently, each sample was associated with an average number of intensity peaks and the average distance between peaks. To investigate the treatment response, the analysis was extended to an equal number (n = 76) of cytokine treatment and control experiments. For cortical actin, Finta was used to evaluate the number of branches, junctions, triple points, quadruple points, end points and the average branch length (then divided by the square root of the normalization factor, 2.17; n = 28 cells). Due to the heterogeneity of the actin staining, the entire field of view, instead of 10 × 10 µm^2^ ROIs, was processed. Next, the data were divided by a normalization factor (calculated as follows: frame area/ expansion factor/ number of cells in the frame). MorphoLibJ plugin (see tubulin) was used to calculate the number and the size of the areas enclosed between branches (termed corrals). The number of corrals was then divided by the frame area (cell area/expansion factor). The average area of corrals was calculated and then divided by the expansion factor, 4.7. The average of the perimeters of corrals was calculated and divided by the square root of the expansion factor: 2.17. For the bleb analysis, an equal number of cells for control and the cytokine-treated samples (N = 93) was analyzed through the observation of the actin-positive cytoplasmic protrusion. This value was then divided by the number of cells in the frame. The average number of blebs per cell was thus calculated. For the morphology analysis of mitochondria, regions with high-density mitochondria were selected, processed by Fiji to improve the SNR (Subtract Background and Gaussian Filter), and analyzed with the plugin MorphoLibJ (input “Object Image”), by setting an opportune tolerance value for each ROI. A binary image was produced and analyzed with the MorphoLibJ tool “Analyze”, obtaining information on the area, perimeter, and circularity of mitochondria. To obtain the dynamical and structural information (iMSD experiments), we employed a custom MATLAB script (MathWorks Inc., Natick, Ma) which computes the spatiotemporal correlation function by Fast Fourier methods. The script is fully described here in^45^. The iMSD curve derived from the script brings out the diffusion law and two diffusion parameters (α, Dm). These are key parameters to describe the diffusive motion: α for granules motion (α < 1 as anomalous diffusion, α = 1 as isotropic diffusion, α > 1 as guided diffusion) and Dm for the diffusivity inside confined regions. A more exhaustive description of the image processing can be found in^45^. For the phasor-FLIM analysis, metabolic maps were produced in Python 3.6 taking into consideration NADH-free and NADH-bound monoexponential characteristic fluorescence lifetime, to enable metabolic measurements in Phasor-FLIM. Indeed, our procedure adheres to what described in Ref.^34^ with the following exception: letting the algorithm sample the phasor plot to automatically map metabolism ranges. In fact, the most extreme intersection points between the experimental point cloud and the straight line joining NADH-free and NADH-bound reference points can be determined geometrically. The next step is computing the distance between each data point and the line perpendicular to the metabolic segment passing through one of the two intersection points. The maps’ look-up tables come from linear segmented colormaps setting 10% and 90% values to anchor the colormap limits. Mann–Whitney test and Shapiro–Wilk normality test were performed for the statistical analysis. For the normally distributed data we choose the unpaired t test, instead for the data not normally distributed we choose the Kolmogorov–Smirnov test (nonparametric) (iMSD analysis). P-value < 0.05 for statistical significance.

### Supplementary Information

Supplementary Information.

## Data Availability

Data will be made available upon request to the corresponding author, Francesco Cardarelli (francesco.cardarelli@sns.it).
