# Supplementary material for: Unveiling nanoscale optical signatures of cytokine-induced β-cell dysfunction
Source: Sci Rep. 2023 Aug 16;13:13342. doi: 10.1038/s41598-023-40272-9 (PMC10432522; doi:10.1038/s41598-023-40272-9)
Supplement: Supplementary file 1 — Supplementary Information. [file 41598_2023_40272_MOESM1_ESM.docx]

**Supplementary Figures**


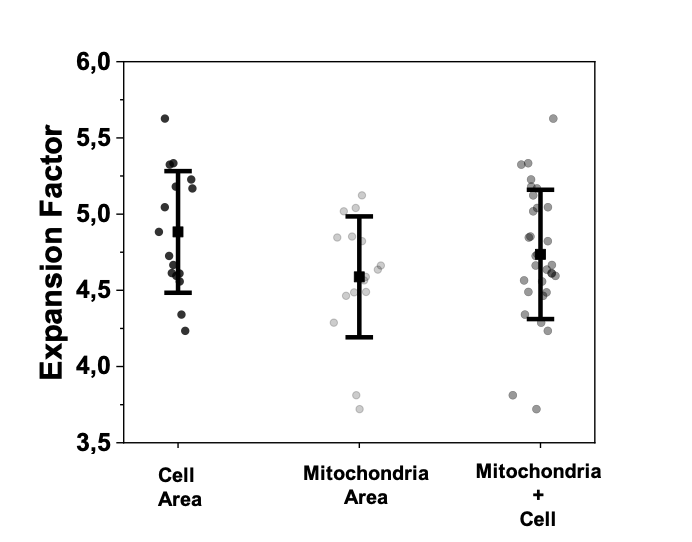


**Fig. S1. Expansion factor.** The EF characterization was performed using subcellular (mitochondria) and cellular (actin staining) in order to achieve a more accurate EF (mitochondria + cell area EF: 4.7±0.43 SD). The number of examined cells was 30 (15 for actin staining and 15 for mitochondria analysis).


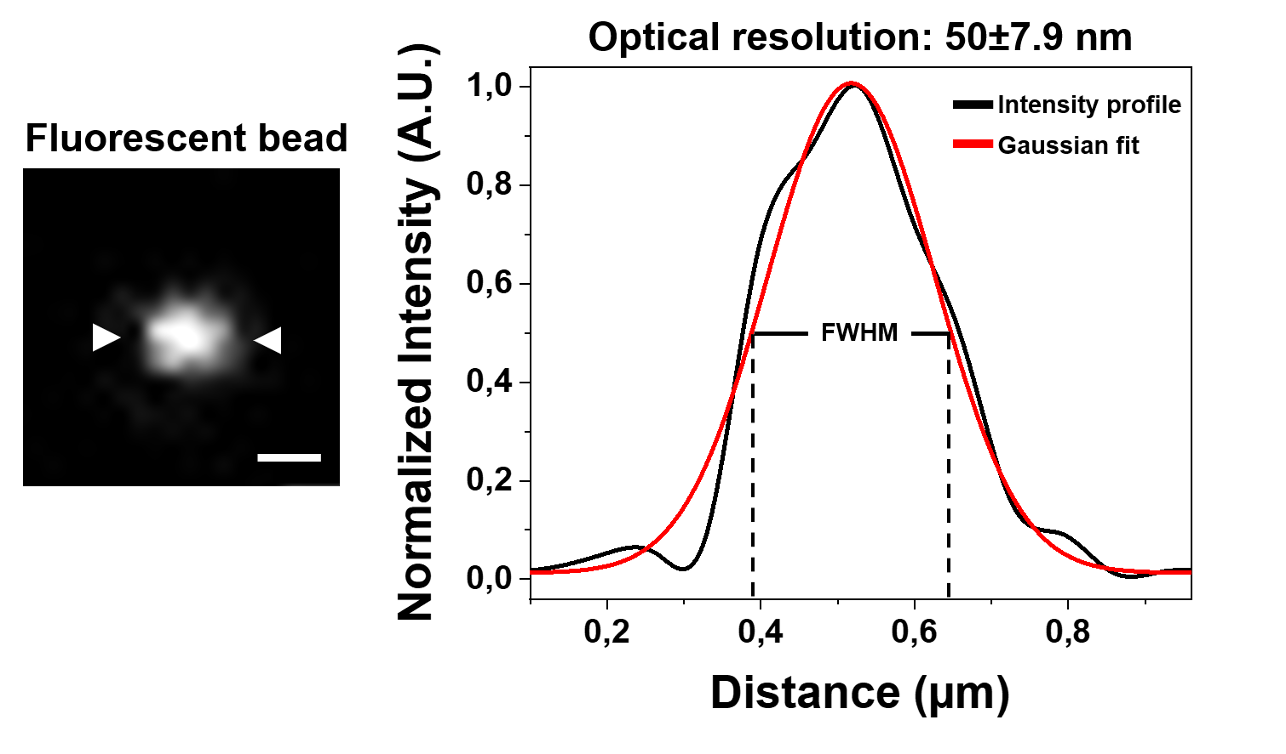


**Fig. S2. Optical resolution.** Confocal images of 100 nm fluorescent beads (TetraSpeck Microspheres, Invitrogen, T7279) to determine the optical resolution. The intensity profiles were fitted to a 2-D-Gaussian function to calculate the FWHM for individual beads shows (n. of beads= 3). Exc. 488 nm. Scale bar 0.3 µm.


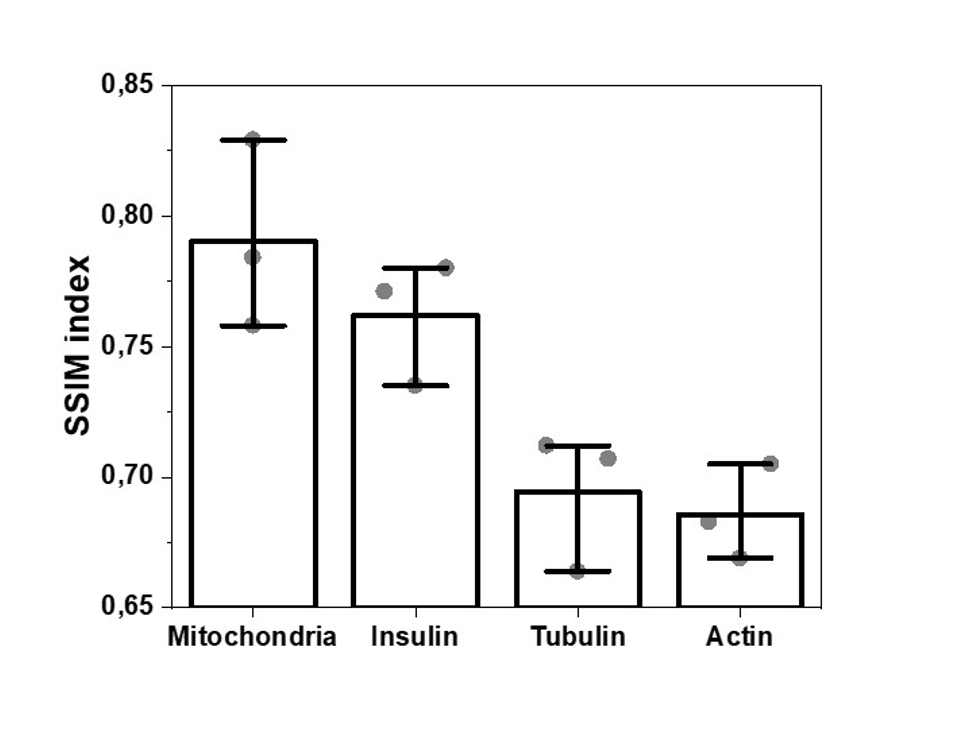


**Fig. S3. Structural similarity.** Comparison of the structural similarity between two confocal images using the structural similarity index measure (SSIM index; range value between -1 and 1; two identical images have an SSIM index close to 1). The data were shown as the mean ± SD (n=3 for each stained structure).


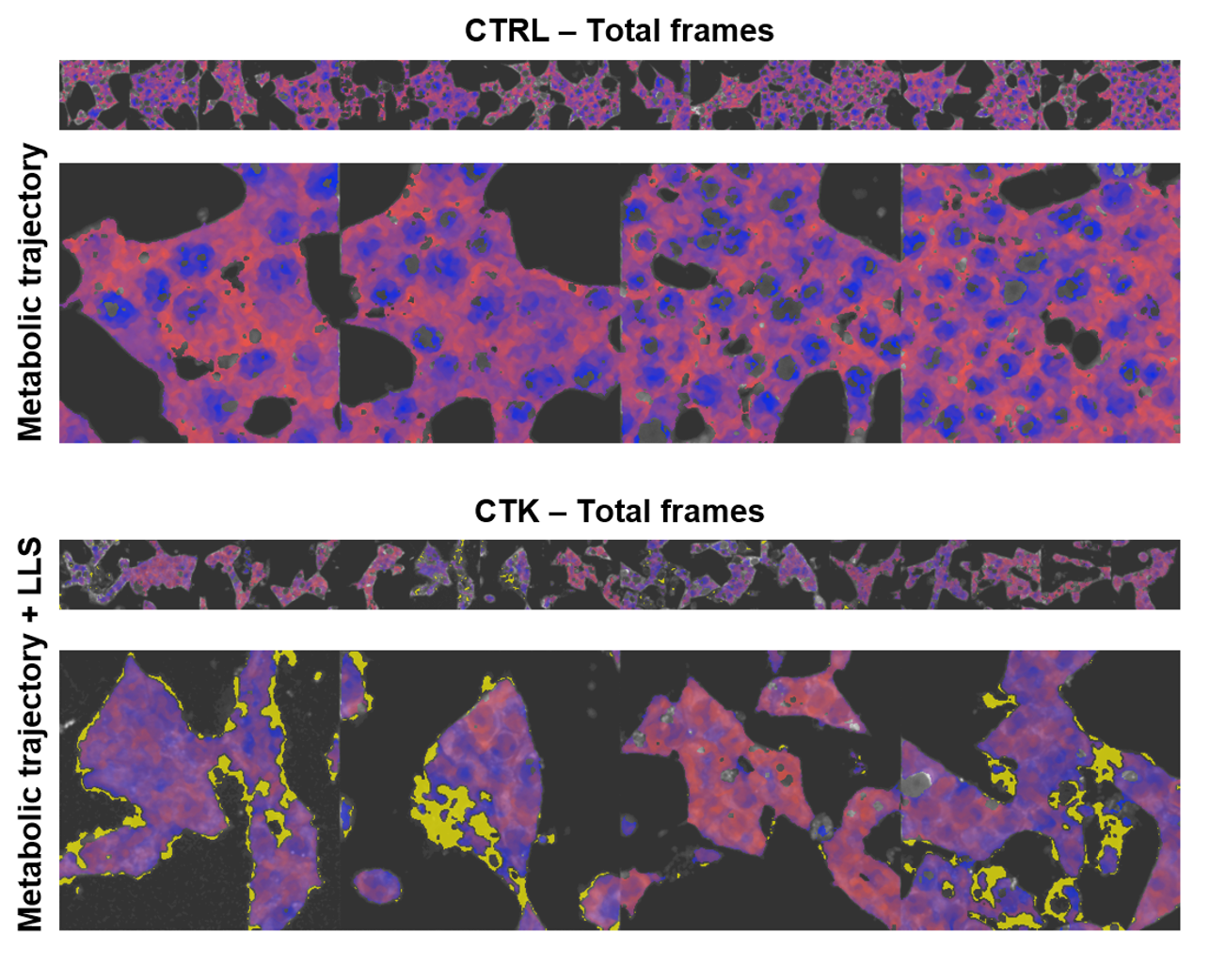


**Fig. S4. Phasor-FLIM signature of metabolic stress.** Cytokine-treated samples show high pixel numbers characterized by long lifetime signature (yellow) respect with the control sample.


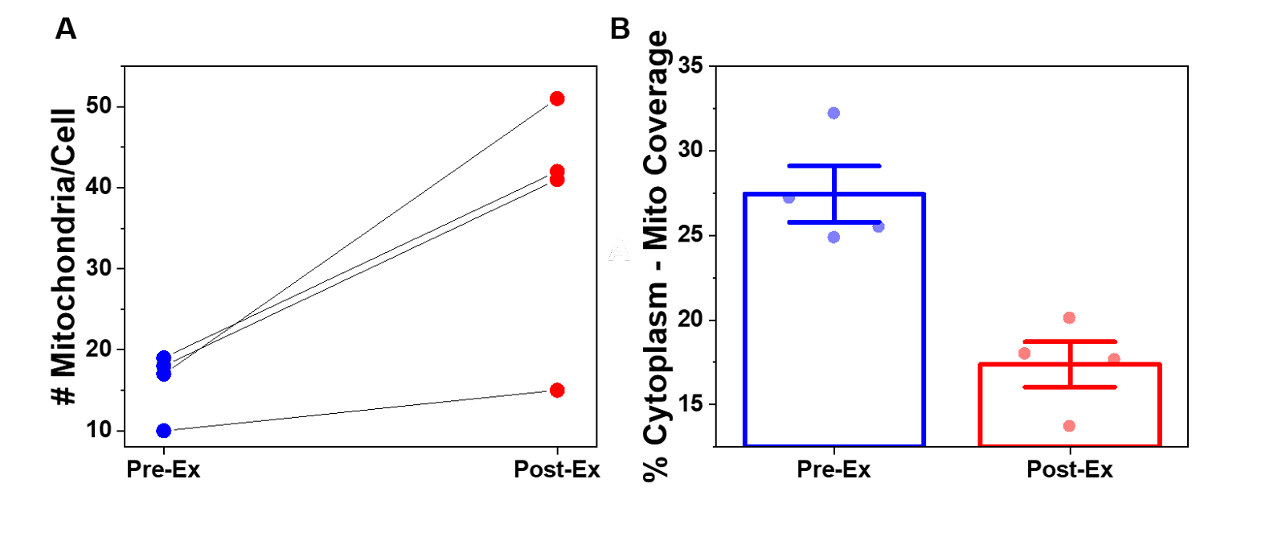


**Fig. S5. Mitochondria count and coverage analysis.** **A.** The number of mitochondria per cell in pre-(blue circle) and post-expansion samples (red circle) (N = 4 cells). **B.** The average percent cytoplasm area (cytoplasm area - nuclear area) which contains mitochondria.


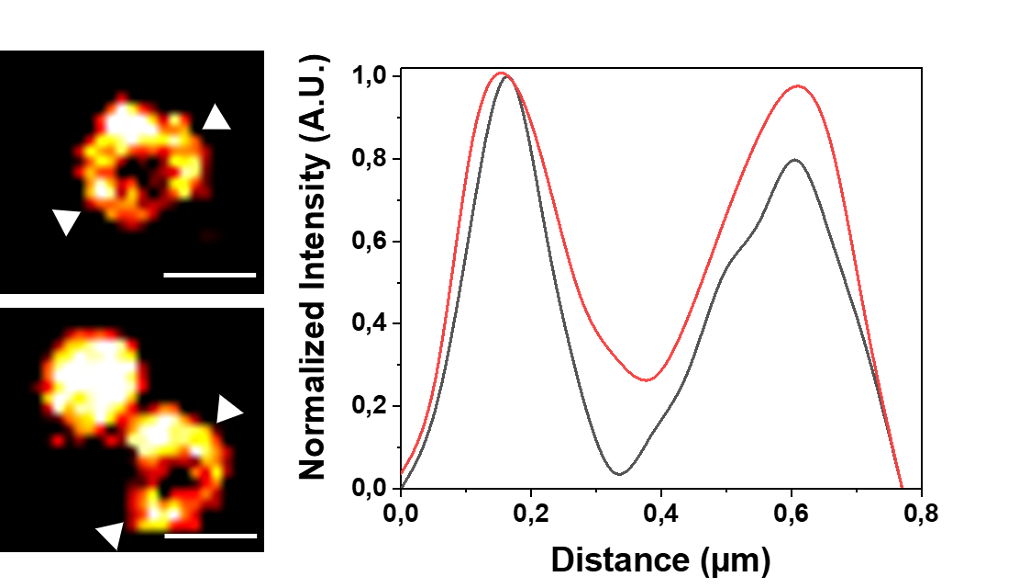


**Fig. S6. Insulin granule expansion.** Profile of the intensity of expanded insulin granules. The crystalline structure of insulin shows a doughnut-shape caused by the isotropic expansion of the labeled granules. Scale bar 0.5 µm.

**Fig. S7. Uncropped Western Blot**


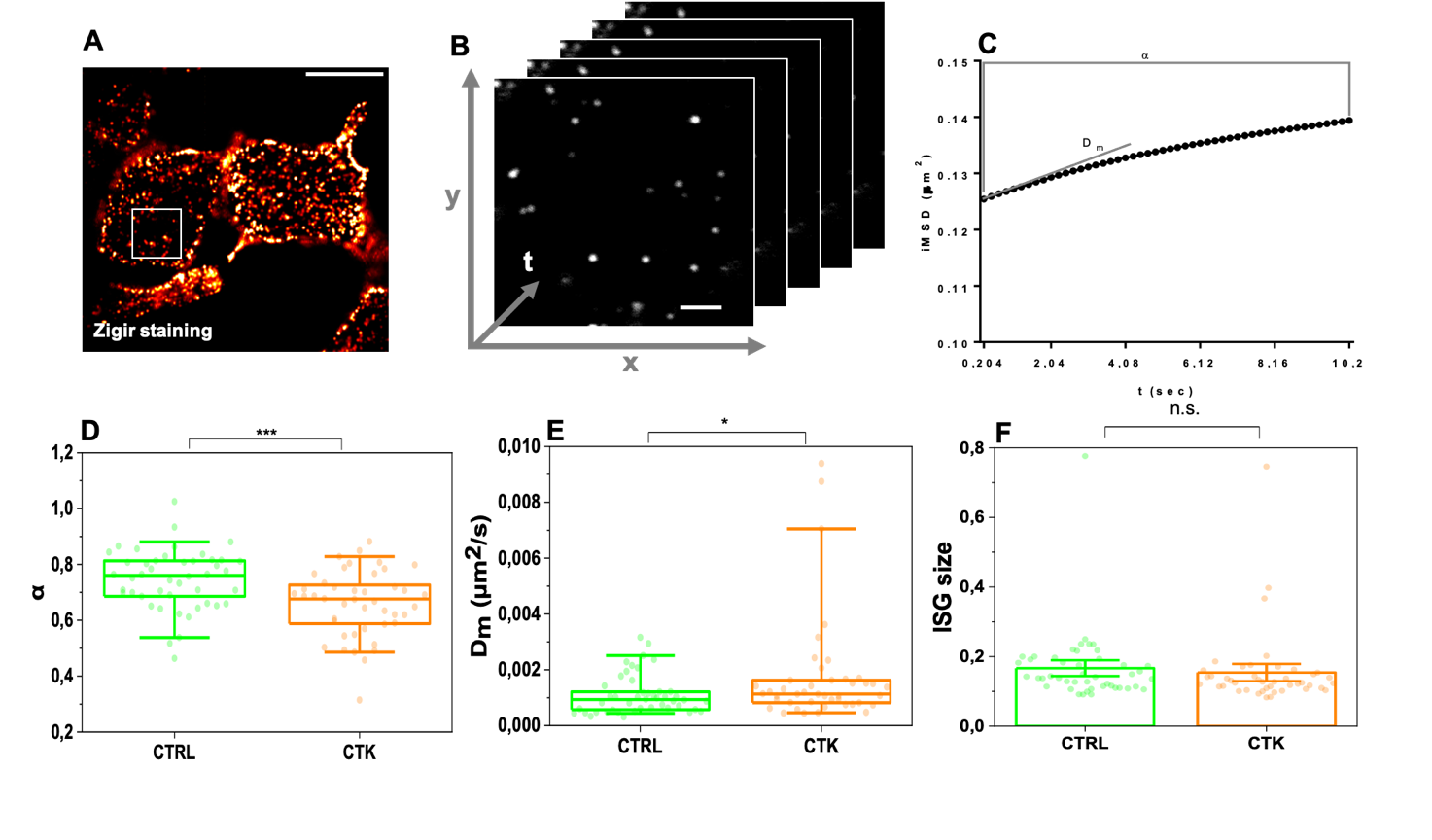


**Fig. S8. iMSD explanatory figure**: **A.** Cells marked by ZIGIR fluorophore were imaged (2x image scale bar 10 μm) with 500 frames time-lapse images **B.** Example of a stack of images acquired at 204.80 ms per frame. Scale bar 2 μm. **C.** iMSD analysis pointed out a granule motion descriptor (α) and diffusivity coefficient (Dm) for each time-lapse. **D.** Comparison of the α parameter between control (CTRL, n = 46) and cytokines treatment (CTK, n = 44) (3 independent experiments). **E.** Comparison for the diffusion coefficient (Dm) between CTRL (n = 46) and CTK (n = 44) (3 independent experiments). We performed the Shapiro-Wilk normality test, unpaired t test for α and Kolmogorov-Smirnov test for Dm based on the normality (see Materials and Methods). **F.** Average size of ISG labeled with Zigir is not affected by cytokines treatment, shown as bar ± SE. A Mann–Whitney test was performed (n.s.= the two distributions are not significantly different).


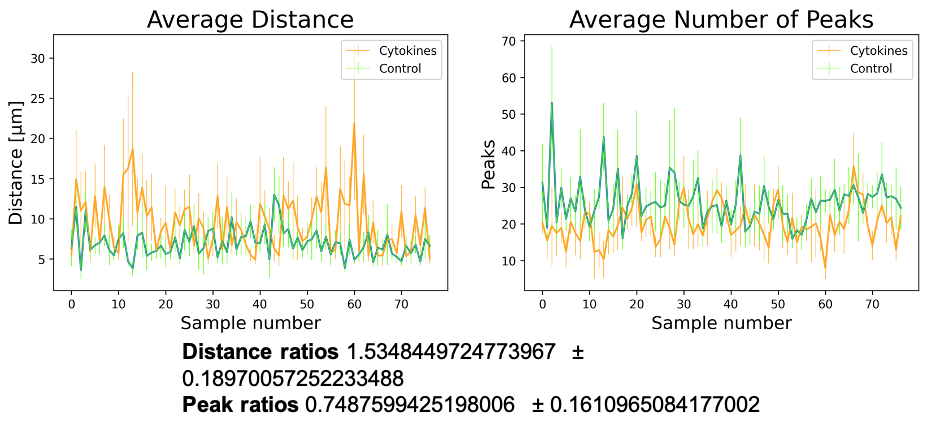


**Fig. S9. Intensity distribution analysis of MT mesh.** Example of the intensity plot profiles to evaluate the effect of cytokine treatment on each sample in terms of microtubule branching. The figure shows the average number of intensity peaks and the average distance between the peaks themselves, alongside standard deviation.

**Fig. S10. Western blot analysis.** Western blot analysis of tubulin (**A**) and actin (**B**). The differences were not statistically significant. n=5 independent experiments.


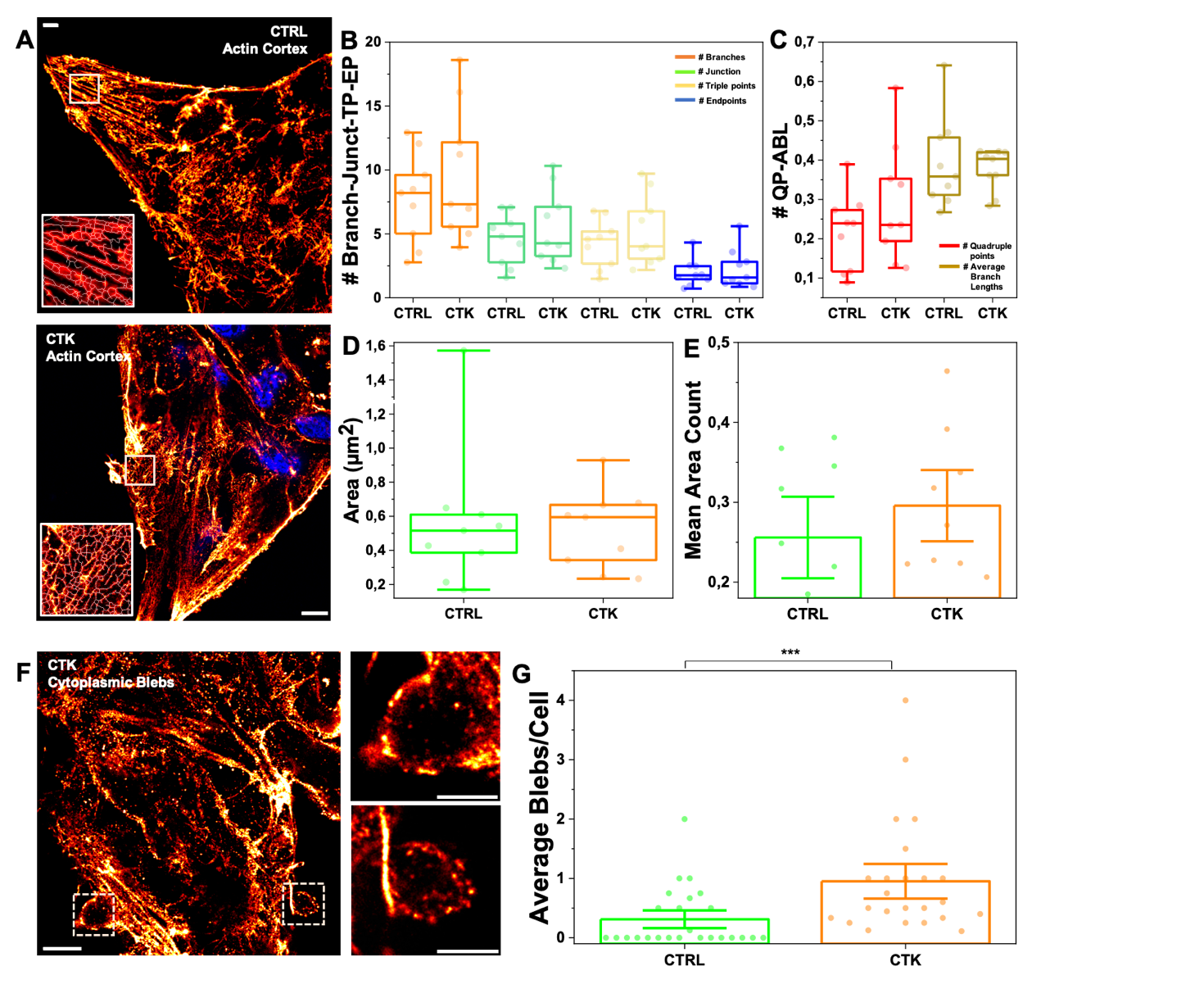


**Fig. S11. Super-resolution images of cortical actin do not show significant structural alteration. A.** Representative images of the actin meshwork in expanded INS-1E cells untreated (control, CTRL) or treated with cytokines (CTK). Cells were stained for Phalloidin and DAPI and acquired by confocal microscope using 405 and 488 excitation light, respectively, with 63x/NA1.4 objective lens. The entire cell clusters were then analyzed by FiNTA and skeletonized through Fiji, to quantify the number of branches (Branch), junction (Junct), triple points (TP), endpoints (EP) (**B**), quadruple points (QP) and average length branch (ALB) (**C**). No significant differences in the cortical actin density were highlighted. Data were presented as box plots with whiskers at the 5th and 95th percentiles, the central line at the 50th percentile, and the ends of the box at the 25th and 75th percentiles (n=28; 3 independent experiment). Scale bar 10 µm. (**D**) Cortical actin opening (corrals) analysis and (**E**) corrals count performed by using MorphoLibJ algorithm. Data were presented as box plots with whiskers at the 5th and 95th percentiles, the central line at the 50th percentile, and the ends of the box at the 25th and 75th percentiles (n=28) (**D**); Bar ± SEM (M) shows the number of corrals. **F.** Representative image showing pathological blebs in cytokines treated sample and bleb count analysis (**G**), shown as bar ± SD (n=93). A Mann–Whitney test was performed (***P < 0.001).


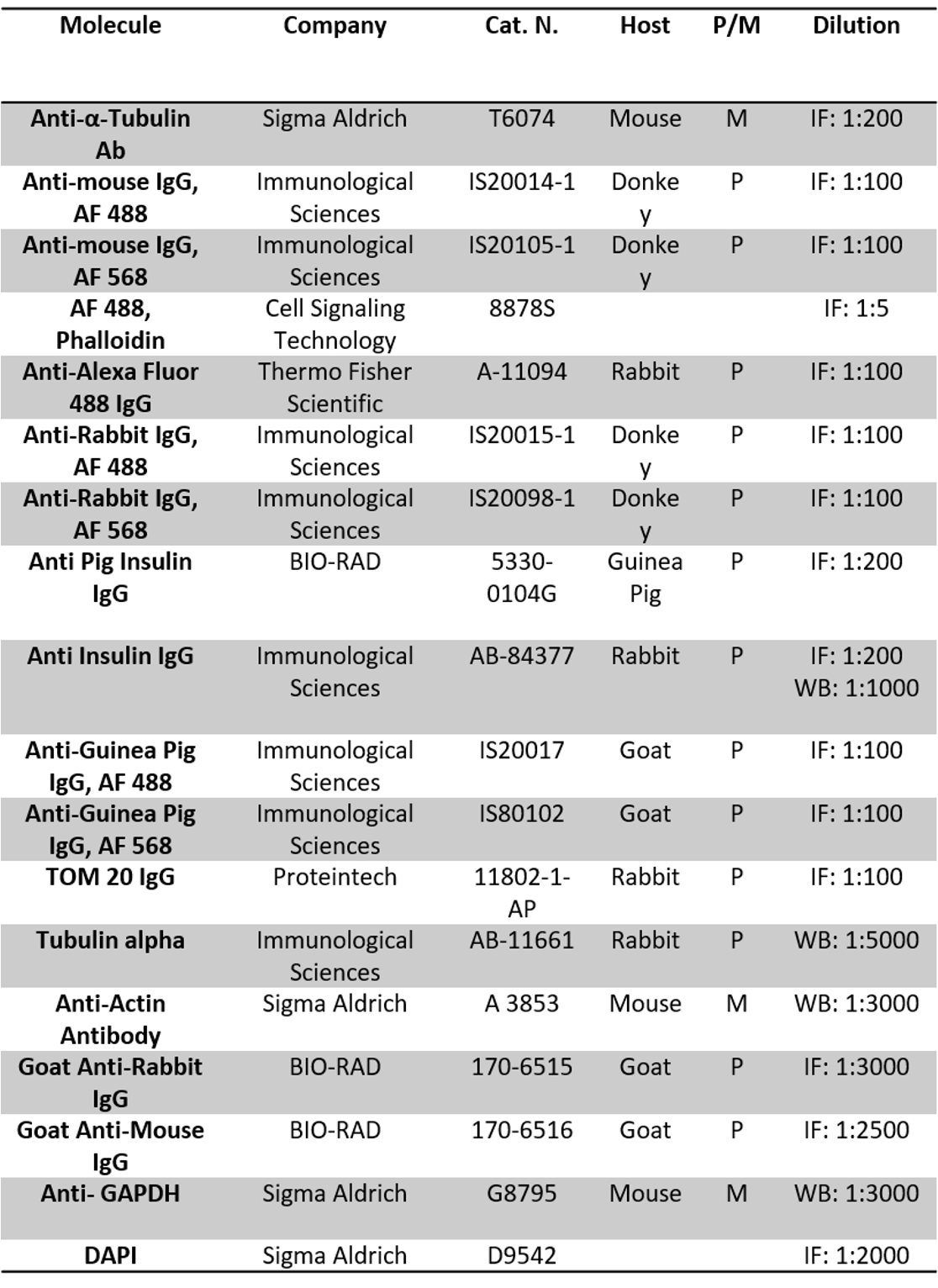


**Table S1.** Antibodies and fluorescent dyes used for immunofluorescence (IF) and for Western Blot (WB), with their dilution.
